# Bio-activating ultrafine grain titanium: RNA sequencing reveals enhanced mechano-activation of osteoconduction on nanostructured substrates

**DOI:** 10.1371/journal.pone.0237463

**Published:** 2020-09-24

**Authors:** Rebecca A. Reiss, Terry C. Lowe, Johnny A. Sena, Oleg Makhnin, Melanie C. Connick, Patrick E. Illescas, Casey F. Davis

**Affiliations:** 1 Biology Department, New Mexico Institution of Mining and Technology, Socorro, New Mexico, United States of America; 2 George S. Ansell Department of Metallurgical and Materials Engineering, Colorado School of Mines, Golden, Colorado, United States of America; 3 National Center for Genome Resources, Santa Fe, New Mexico, United States of America; 4 Mathematics Department, New Mexico Institute of Mining and Technology, Socorro, New Mexico, United States of America; University of Vermont, UNITED STATES

## Abstract

Titanium is essentially absent from biological systems yet reliably integrates into bone. To achieve osseointegration, titanium must activate biological processes without entering cells, defining it as a bio-activating material. Nanostructuring bulk titanium reduces grain size, increases strength, and improves other quantifiable physical properties, including cytocompatibility. The biological processes activated by increasing grain boundary availability were detected with total RNA-sequencing in mouse pre-osteoblasts grown for 72 hours on nanometrically smooth substrates of either coarse grain or nanostructured ultrafine grain titanium. The average grain boundary length under cells on the conventional coarse grain substrates is 273.0 μm, compared to 70,881.5 μm for cells adhered to the nanostructured ultrafine grain substrates; a 260-fold difference. Cells on both substrates exhibit similar expression profiles for genes whose products are critical for mechanosensation and transduction of cues that trigger osteoconduction. Biological process Gene Ontology term enrichment analysis of differentially expressed genes reveals that cell cycle, chromatin modification, telomere maintenance, and RNA metabolism processes are upregulated on ultrafine grain titanium. Processes related to immune response, including apoptosis, are downregulated. Tumor-suppressor genes are upregulated while tumor-promoting genes are downregulated. Upregulation of genes involved in chromatin remodeling and downregulation of genes under the control of the peripheral circadian clock implicate both processes in the transduction of mechanosensory information. Non-coding RNAs may also play a role in the response. Merging transcriptomics with well-established mechanobiology principles generates a unified model to explain the bio-activating properties of titanium. The modulation of processes is accomplished through chromatin remodeling in which the nucleus responds like a rheostat to grain boundary concentration. This convergence of biological and materials science reveals a pathway toward understanding the biotic-abiotic interface and will inform the development of effective bio-activating and bio-inactivating materials.

## Introduction

### Titanium as a bio-activating material

The compatibility of titanium (Ti) with bone is established by nearly seven decades of research, but the molecular basis for this property is not well understood. The term ‘biocompatibility’ refers to interaction of the immune system with implanted materials but is an archaic term that doesn’t account for molecular level processes induced by specific physical characteristics of abiotic substrates. Nanostructuring Ti by methods such as Continuous Equal Channel Angular Pressing (C-ECAP) introduces molecular level microstructural features which increase strength by 30% to 100%. Grain boundary misorientations angles, energies, and total lengths per unit volume increase due to C-ECAP reducing grain size from an average of 20 μm (coarse grain or CG) to less than 0.15 μm (ultrafine grain or UG) [1]. This alteration of microstructure not only changes the physical characteristics, it has biological consequences. There are multiple lines of evidence that verify osteogenic cells respond differently to CG- and UG-Ti, with cells grown on UG substrates exhibiting more rapid attachment, proliferation, and differentiation [2]. However, questions remain, including how do cells sense the difference between CG and UG substrates and how does this affect cell proliferation? Total RNA sequencing (RNA-seq) techniques can interrogate the genome-level processes that are activated by contact with grain boundaries. The differential growth of cells on these substrates indicates an underlying difference in the ability to activate biological processes involved in cell proliferation, hence we propose the updated term “bio-activating” to designate a specific aspect of “biocompatibility.” This distinguishes the effect of bulk materials used for implants from nanoparticles that are labeled as ‘bioactive.’ In pharmacology ‘bioactive’ assumes the material enters the cell and participates in cell metabolism. While a thorough understanding of the bio-activating properties of bulk materials will facilitate improved implant design, eventually it will also provide insight and potential targets for materials with the opposite effect; bio-inactivating materials that can sequester and inactivate bacteria and viruses.

### Mechanotransduction: Cell attachment

Mechanosensation and transduction describe the initial interaction of a cell with a substrate and subsequent response [3]. Mechanosensation experiments typically involve carbon-based tissues and polymers with stiffness magnitudes associated with biological material, which varies from about 0.2 kPa for brain tissue to 50 kPa for pre-calcified bone [4]. Bone elastic moduli range from about 10 to 25 GPa depending upon the type [5]. Ti elasticity is in the range of 80 GPa to 110 GPa, four to ten times stiffer than the bone into which it is implanted and contains insignificant amount of carbon.

Integrins present in the extracellular matrix (ECM) are the first to contact a surface, which initiates outside-in signaling, transmitting the force to other protein complexes of the cytoskeleton. Inside-out signaling results in the development of focal adhesions necessary for cell adherence [6]. Primary cilia are also involved in mechanosensing from abiotic substrates [7]. Caveolae (‘little caves’) are membrane invaginations lined with the caveolin-1 protein (Cav1) that sense mechanical stress in the membrane [8].

It is increasingly evident that the nucleus plays a critical role in the mechanosensory pathway [9]. The Linker of Nucleoskeleton and Cytoskeleton (LINC) protein complex transmits the mechanosensory signal through the nuclear envelope to lamins, proteins that line the interior of the nucleus [10]. Lamin A interacts with a nuclear-localized form of Vascular Endothelial Growth Factor A (VegfA), which can regulate the fate of mesenchymal stem cells [11]. Lamins also interact with Polycomb Group (PcG) proteins, which are involved in epigenetic remodeling that results in gene expression changes [12].

The reduced grain size in UG-Ti changes surface characteristics such as surface energy [13]. The specific characteristic(s) to which cells respond remains unresolved. However, the ability to fabricate substrates with quantifiable differences in physical characteristics combined with RNA-seq provides an opportunity to probe the bio-activating properties of Ti.

### Osteogenesis: Cell proliferation and differentiation

The successful integration of an implant into bone is dependent upon bone healing processes and requires the presence of mesenchymal stem cells (MSCs) that can differentiate into preosteoblasts when provided appropriate environmental cues. The first stage of osteogenesis, osteoinduction, is the induction of MSCs to differentiate into preosteoblasts. Adherence, growth, and proliferation on a substrate constitute osteoconduction. As cells reach confluence, osseointegration can proceed, in which preosteoblasts differentiate into mineralized implant-adhering osteoblasts [14].

Studies of signaling proteins during osteogenesis indicate VegfA, Insulin-like growth factor 1 (Ifg1), and leukemia inhibitory factor (Lif) play important roles, however, the relationship of these growth factors is complicated and remains to be fully elucidated [15, 16]. Chromodomain helicase DNA binding protein 9 (Chd9) is a chromatin re-modeling protein that binds to bone-specific promoters in osteogenic cells [17].

Although the existence of the circadian rhythm was first established in mammalian response to light/dark cycles that involve gene expression cycling in the hypothalamus, it is now established that most cells have circadian clocks, known as peripheral clocks and that these are linked to cellular processes. Osteogenic cells and bone remodeling are no exception, peripheral clock gene expression is an integral part of osteogenic processes [18].

Transcript expression profiles for six genes in mouse preosteoblast MC3T3- E1 cell lines established using Northern analysis are indicators of cell identity [19]. Transcripts for pro a2 (I) collagen (a.k.a. collagen type I alpha 2, or *Col1A2*) are abundant in preosteoblasts as is the collagen protein. For subclone 4 grown in non-mineralizing conductions, osteoblast-specific Transcription Factor 2 (*Osf2/cbfa1)* (a.k.a. runt related transcription factor 2 or *Runx2*) is expressed at a low level while alkaline phosphatase (a.k.a. alkaline phosphatase liver/bone/kidney or *Alpl*) is barely detectable. Not detectable by Northern analysis are bone sialoprotein (a.k.a. integrin binding sialoprotein or *Ibsp*), osteocalcin (a.k.a bone gamma-carboxyglutamate protein *2* or *Bglap2*), or parathyroid hormone receptor (a.k.a. the parathyroid hormone 1 receptor or *Pth1r*) transcripts.

Non-coding RNAs (ncRNA) are recently recognized effectors of cell function. Long ncRNAs (lncRNA) are greater than 200 bp. Metastasis associated lung adenocarcinoma transcript 1 (*Malat1)* lncRNA promotes osteogenesis during bone regeneration [20]. MicroRNAs are short (about 22 nt) RNAs that regulate the availability of transcripts. MicroRNA 17hg (*Mir17hg*) is a region that produces six miRNAs that are all required for normal skeletal development [21]. Although *Ftx* is best known as a long noncoding RNA that acts an effector of X-chromosome silencing in female embryos, it also functions as an oncogenic miRNA sponge in multiple cancer types, including osteosarcoma [22]. In the mouse genome, circular RNAs (ciRNAs) are associated with the *Ftx* region [23, 24]. The circular configuration of circRNAs makes them resistant to degredation in the cytoplasm and are important regulatory molecules whose functions we are just beginning to understand [25]. An interesting example of a circRNAs in osteogenesis is the competition between a circRNA and Osteoglycin (*Ogn*) for an miRNA associated with osteoporosis [26]. Piwi-interacting RNAs (piRNAs) are 21 to 35 nucleotides segments of RNA that can orginate from lncRNAs and are active during development [27]. These are also found in extracellular vesicles (EV), including those from multiple meloma bone disease that are capable of inhibiting osteogenesis [28].

The potential of transcriptomics to advance the discipline of biomaterials has already been recognized, although most experiments involve microarrays, which require an *a priori* knowledge of genes to be assayed [29]. RNA sequencing (RNA-seq) is a more expensive option, but it has the advantage of facilitating new discoveries about gene expression and has been applied to the osteoinductive properties of calcium phosphate ceramics [30]. Traditional complementary DNA (cDNA) libraries made from the poly-adenylated (Poly-A) fraction of RNA provide information on protein coding transcripts. Ribosome-depleted RNA libraries sequencing retain both protein coding RNAs and some ncRNAs, providing a glimpse into the roles that these diverse molecules play.

The objective of this research project is to determine if RNA-seq can detect gene expression changes associated with the response of preosteoblasts during the osteoconduction stage on nanometrically smooth, compositionally identical substrates that differ by only their crystallographic structure. The substrates have an average grain size of 10.9 +/- 7.3 μm (CG) and 0.24 +/- 0.38 (UG), a 45-fold difference [31]. Characterization of mouse preosteoblasts growth on these discs included the development of a new bio-physical parameter, the ratio between the average grain boundary length (GBL) per unit area to the average cell contact area covered by cells (R_GBL/cell_). After 72 hours not only are there 3-fold more cells on UG-Ti, the cells are larger, with an average cell covering about 5,612 μm^2^ on UG and 1,457 μm^2^ on CG-Ti. The average grain boundary length under a cell on CG-Ti was 273.0 μm, compared to 70,881.5 μm for UG-Ti; a 260-fold difference. We analyzed total RNA from mouse pre-osteoblasts grown on these nanometrically smooth UG- and CG-Ti substrates for 72 hours to elucidate the expression of protein coding and non-coding RNAs.

## Materials and methods

### Ti disc preparation

Details of disc fabrication and their physical properties are published elsewhere [31] and are available in the Compendium of Biomaterials Transcriptomics (cBiT) [32]. Briefly, CG-Ti rods were obtained from the Carpenter Technology Corporation (Reading, Pennsylvania), UG rods were bulk nanostructured via C-ECAP, applying at least four passes with interpass billet rotations of 90° following route B_c_ at 200°C to 350°C. One mm thick discs were cut from rods 13.5 mm in diameter. To reduce the effect of surface topography on cell growth, all discs were polished to a <2 nm average surface roughness (R_a_). To facilitate handling the discs with sterile forceps (Miltex 6–120, Germany), a 2 mm flat section was ground on diametrically opposed edges of the otherwise circular discs.

Ti disks were sterilized with isopropanol and placed in 24-well cell culture dishes plates not treated for tissue culture (USA Scientific, CC7672-7524) to reduce the number of cells attached to the plates rather than the Ti.

### Preosteoblast culture

**Fig 1:**
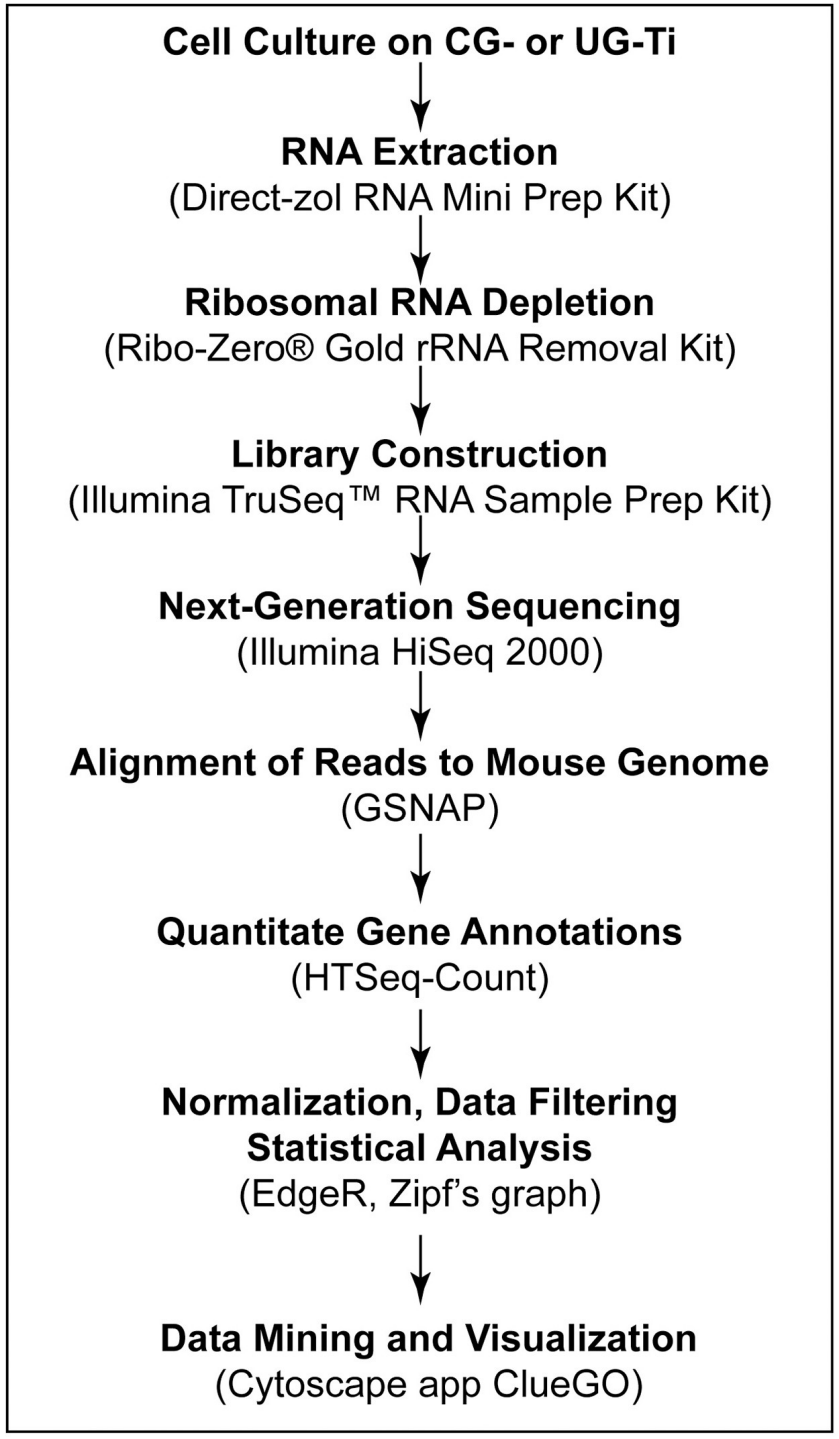
Bioinformatic workflow. Workflow used to generate network analysis. Kits, software and instrumentation used to generate data for each step are shown in parentheses.

The workflow (Fig 1) begins with the culture of the Murine preostoeblast cell line, MC3T3-E1 sub-clone 4 (American Type Culture Collection (ATCC), Rockville, MD) in Alpha Minimum Essential Medium GIBCO, Grand Island, NY) supplemented with 10% fetal bovine serum (FBS, GIBCO, Grand Island, NY) and 1% penicillin/streptomycin (Cellgro, Manassas,VA). In order to focus on the early, non-mineralizing phenotype at 72 hours, the media was not supplemented with acetate. Each disk was seeded with 4 x 10^4^ and plates were incubated at 37°C with 5% CO_2_. After 72 hours, the Ti discs were moved to a culture disc containing trypsin, taking care not to disturb the cells. This step further minimized the risk of isolating cells adhered to the plates. Cells from four discs were pooled, centrifuged at 250 x G for 5 min, the resulting pellet was resuspended in RNA Later Reagent (Sigma-Aldrich, T9424), and stored at -80°C.

### RNA extraction

Total RNA was extracted from cell pools using the Direct-zol RNA Mini Prep kit standard protocol (Zymo Research Corp., R2050) and included the treatment of the RNA with DNase I to eliminate genomic DNA. RNA concentrations and quality were determined using a Nanodrop 2000 UV-Vis Spectrophotometer (Thermo Scientific) and were confirmed with the Qubit RNA HS assay kit (ThermoFisher Scientific, Q32852). RNA size and integrity were measured using a 2100 Bioanalyzer (Agilent Technologies, Santa Clara, CA). The library input material had RNA Integrity Numbers (RINs) ranging from 7.3 to 8.6.

### Library construction and sequencing

Library preparation and sequencing was performed at the National Center for Genome Resources (NCGR, Santa Fe). Prior to library preparation, cytoplasmic and mitochondrial ribosomal RNAs (rRNAs) were removed from the samples using the Ribo-Zero^®^ Gold rRNA Removal Kit (Illumina, San Diego). RNA-depleted libraries were prepared following the Illumina TruSeq^™^ RNA Sample Prep Kit v2 (Illumina, San Diego), which produced inserts sized from 120 to 200 bp, with an average of 150 bp. Unique indexes were added to each sample during library construction to allow multiple libraries to be pooled into a single lane of the Illumina flow cell. Libraries were validated using Nanodrop quantification and Bioanalyzer 2100 analysis to determine size and quantity. The libraries were run in a single lane of a flow cell on an Illumina HiSeq 2000 following manufacturer’s protocols to generate single-end 50 bp reads.

### Bioinformatics workflow

#### Data management and analysis

FastQ files generated by Illumina were uploaded to NCGR to facilitate the development of a bioinformatics workflow with a variety of tools, including a data management system for storing and tracking analyses. The reads were subjected to quality control analysis using Fast QC [33]. Reads were then aligned to the mouse reference genome (GRCm38) [34] using the Genomic Short-read Nucleotide Alignment Program (GSNAP), producing binary sequence alignment map (.bam) files [35]. These files are available through Gene Expression Omnibus [36] and ArrayExpress [37]. HTSeq-count was used to generate read counts per gene [38].

Profiles of protein coding genes are expected to align uniformly to exons, whereas ncRNAs can exhibit expression of short regions anywhere in the genome. To verify that read count profiles fit the gene model (ncRNA or protein coding) described by annotations, .bam files were inspected using the Integrated Genome Viewer (IGV) software package [39]. Transcripts that uniformly covered exons were assumed to be coding RNAs. Long ncRNAs are greater than 200 nucleotides in length that can be transcribed from any region, including those that code for protein. Reads that only align to a small section of a gene are indicative of ncRNAs. It is a recent revelation that ncRNAs are common in genomes, so annotation of these important molecules has lagged behind that of protein coding regions [40].

Read count files were normalized and statistically analyzed with empirical analysis of digital gene expression data in R (edgeR) [41]. Cells grown on CG-Ti were used as the controls whereas cell grown on UG Ti were used as the treatment group. The statistical analysis of differential gene expression used the expression on UFG-Ti as the experimental value and compared to CG-Ti. The statistical package edgeR outputs two statistical values per gene; the p value and a value adjusted for a False Discovery Rate of 0.05 (FDR or p(adj)). A statistical issue with large datasets is that at p≤0.05 there is the potential for a large number of false positives, so an p(adj) ≤ 0.05 is considered a more reliable statistical cutoff. However, neither of these values can evaluate the biological significance of any change in gene expression. Gene products work in concert to modulate biological processes, so additional levels of statistical analysis are required to extract meaningful patterns of gene expression changes.

The output of statistical analysis was opened with JMP version 11 (SAS, Cary, NC) for data filtration. Zipf’s power law is used to model complex phenomena [42], has been employed to filter low counts from environmental metagenomic shotgun sequence data [43], and was demonstrated recently to be an effective method of normalization for RNA-seq data [44]. Here it is used to select annotations with adequate read coverage for comparative analyses. The statistical package edgeR calculates log_10_ counts per million (CPM), this was used to rank the genes and the log rank was graphed against log CPM, to produce a power law graph. Genes with read counts near to or above the downturn in the slope are assumed to have adequate read counts for comparative analyses, while the data below the downturn are filtered out. This provides a low-count filtering method and eliminates filtering based on an abritrary the fold-change level.

#### Data mining and visualization

Differentially expressed genes (p≤0.05) were selected to assess biological significance using enrichment analyses, and network visualization techniques with Cytoscape v3.4.0 [45]. The Cytoscape application ClueGO + CluePedia v2.3.2 combines enrichment analysis of Gene Ontology (GO) terms of a gene list with network visualization [46]. A gene product with known functions is assigned Gene Ontology (GO) terms that describe the cellular location(s), molecular function(s), and biological process(es) in which it is involved. For this project, biological processes (BP) GO terms were the most relevant. ClueGO utilizes the DAVID (Database for Annotation, Visualization and Integrated Discovery) gene functional classification tool to extract biologically significant process information from a gene list input by the user [47]. Enrichment analyses are based on the total number of genes associated with a term in the genome compared to the number genes in the input list that share the same term. DAVID calculates a EASE (Expression Analysis Systematic Explorer) score, which is a modified Fisher Exact p-value adjusted for multiple comparisons using the Bonferroni correction (p(adj)) [48]. The probability of the enrichment greater than chance for each GO term is evaluated and only terms with p(adj) ≤ 0.05 are retained. For this dataset, GO Biological Process term levels 3–8 were used. Additional parameters specified in the ClueGO analysis are provided in supplemental information (S1 Text). CluePedia was used to add the genes linked to the GO terms enriched in the network. Gene nodes were annotated with differential gene expression values using the style functions available in Cytoscape. To produce a visually informative network, nodes were arranged manually within Cytoscape using centrality measures established for biological networks [49]. Nodes representing GO terms were arranged with closeness parameters in mind so that nodes assigned to the same overview term are stacked. For gene nodes, placement resembled the principle of shortest path betweenness, where genes that are connected to single GO terms are placed close to the term and genes connected to multiple GO term nodes are between those nodes. Networks were imported into Adobe Illustrator for final markup. The GeneCards database was consulted for details of gene function [50]. Homology searches were accomplished with Basic Local Alignment Search Tool (BLAST) [51].

### Quantitative PCR

Preliminary RNA-seq results were a partial guide to choosing genes for Real-Time Quantitative PCR (qPCR) validation; statistical significance and association of the gene products with osteogenesis were both considered. Primer design was accomplished by using RNA-seq data in conjunction with Primer-BLAST [52]. Since actin b (*Actb*) was not differentially regulated in the total-RNA-seq dataset and the same RNA preparations were used for both qPCR and RNA-seq, it was used to normalize the data. Table 1 lists the genes and primers used for validation. PCR was performed with the iScript^®^ one step RT-PCR kit (Bio-Rad) following manufacturer’s protocols. The comparative C_T_ quantitation protocol was implemented on an Applied Biosystems 7500 Real Time PCR instrument. Three replicates of each sample were analyzed using the comparative CT method to determine the 2^-ΔΔCt^ [53].

**Table 1:** qPCR primers.

Gene ID	Name (alias)	Primers
*Ogn*	Osteoglycin (Osteoinductive Factor)	Ogn F TCTGACACAGCAAGCACCAC
		Ogn R CAGGCATGTGGGCATTTCATC
*Omd*	Osteomodulin (Osteoadherin Proteoglycan)	Omd F TCACACAACAAACTGGAAGACA
		Omd R GGTGTACTGGATCAGGAGAAGG
*Igf1*	Insulin like growth factor 1 (Mechano Growth Factor)	Igf1 F TGCTCTAACATCTCCCATCTCTC
		Igf1 R GGTGAAGGTGAGCAAGCAGA
*Igfbp2*	Insulin-Like Growth Factor Binding Protein 2	Igfpb2 F TCTACTCCCTGCACATCCCC
		Igfpb2 R GCTTCCCGGTATTGGGGTTC
*Cenpf*	Centromere Protein F, (Mitosin)	Cenpf F ACCGAGTTTGAGCCAGAAGG
		Cenpf R CGCTGAGCTCTCCTGAACAA
*Actb*	Actin B	ActB F CCACCATGTACCCAGGCATT
		ActB R CAGCTCAGTAACAGTCCGCC

## Results

### Sequencing results: Raw data and filtering

**Fig 2:**
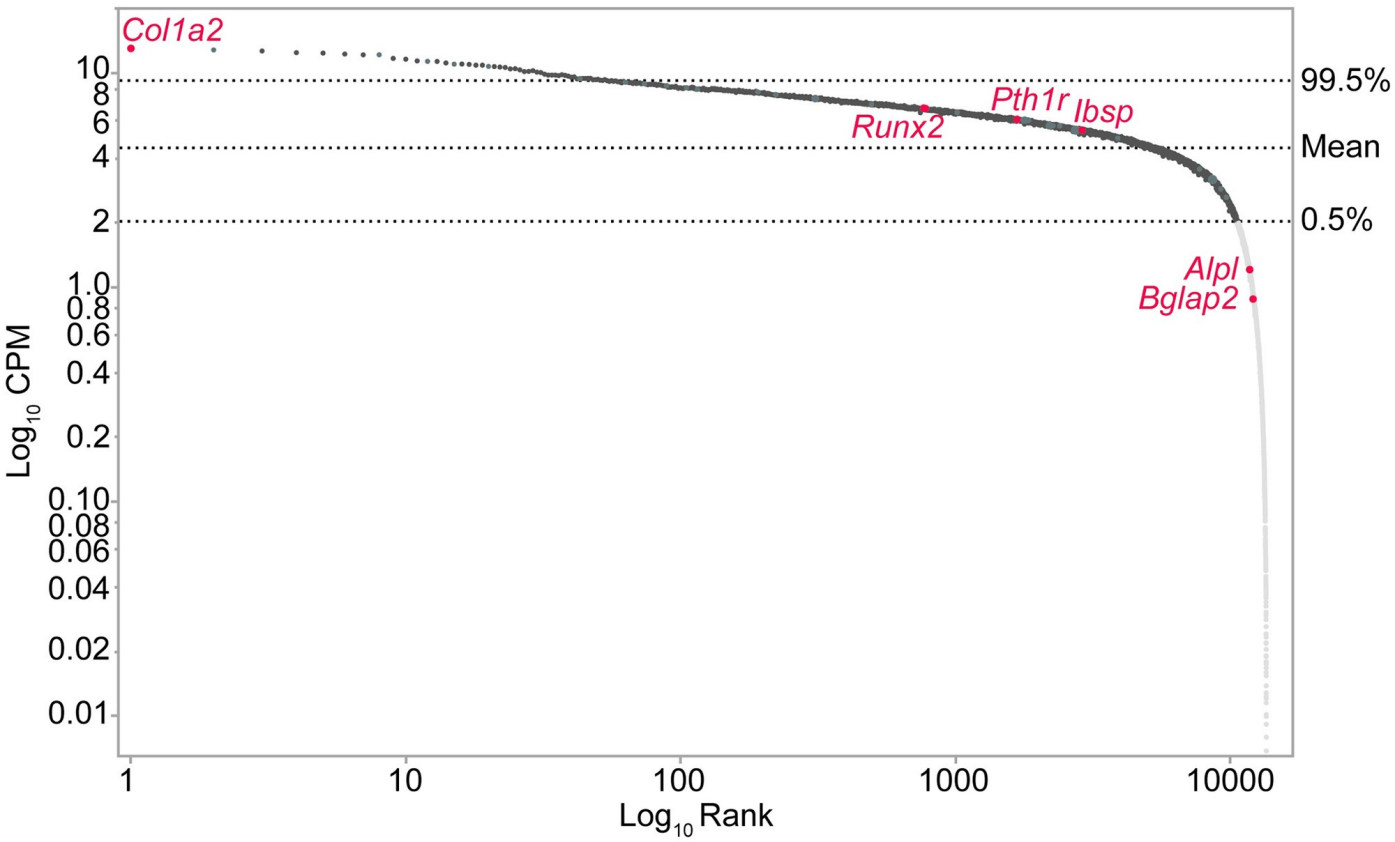
Zipf curve. Dark grey markers are those with adequate coverage to use in comparative analyses, genes shown in light grey were disregarded. Red font indicates genes used as indicators of cell identity. S1 Fig is the same curve annotated with differentially expressed and those of interest in mechanotransduction and osteogenesis.

Raw and processed sequence files are available in the Gene Expression Ominbus [36] and ArrayExpress [37]. The raw data consisted of reads that met stringent quality control requirements (S1 Table in S1 File). The results of the Zipf’s Law plot (Fig 2 and S1 Fig) indicates that transcripts with more than 100 reads (Log CPM = 2) have adequate coverage for further analyses, this included 51.9% out of 20,500 transcripts. Of the 10,633 transcripts, 423 were differentially regulated (p- ≤ 0.05) and six at a p(adj) ≤ 0.05. Of the 423 transcripts, 82.2% were upregulated on UG-Ti.

Transcript abundance using RNAseq of MC3T3-E1 sub-clone 4 compared favorably to the expression profile developed using Northern analysis [19]. None of the six genes were differentially expressed on the two substrates but the CPM is a measure of abundance and can be compared to Northern analyses. *Col1a2* was the most abundant transcript as measured by Northern analysis and by RNAseq, while *Runx2* exhibited intermediate expression in both datasets. *Alpl* had minimal expression in the Northern and was below the statistical cutoff. *Bglap2* was not detectable in the Northerns and was also below the cutoff. *Ibsp* and *Pth1r* transcripts were detected in RNAseq data but not in Northern analysis. It remains to be determined if these differences are attributable to differences in substrate (cell culture plastic or Ti) or to the increased sensitivity of RNAseq.

Other genes involved in mechanosensation and osteogenesis were not differentially regulated (S2 Table in S1 File and S1 Fig). For these processes to occur, the proteins responsible must be available, so differential expression is not expected. For osteogenesis markers, the lack of transcription or differential expression indicates that the cells were still in osteoconduction and had not yet begun to differentiate.

Integrin functions are well established in mechanosensation and there are numerous integrins expressed (S2 Table in S1 File and S1 Fig). *Itga6* is the only integrin gene that was differentially expressed, it is cell cycle regulator linked to apoptotic processes (Fig 4). This transcript was downregulated on UG-Ti and is known to be under control of the circadian clock [54].

### qPCR results

**Fig 3:**
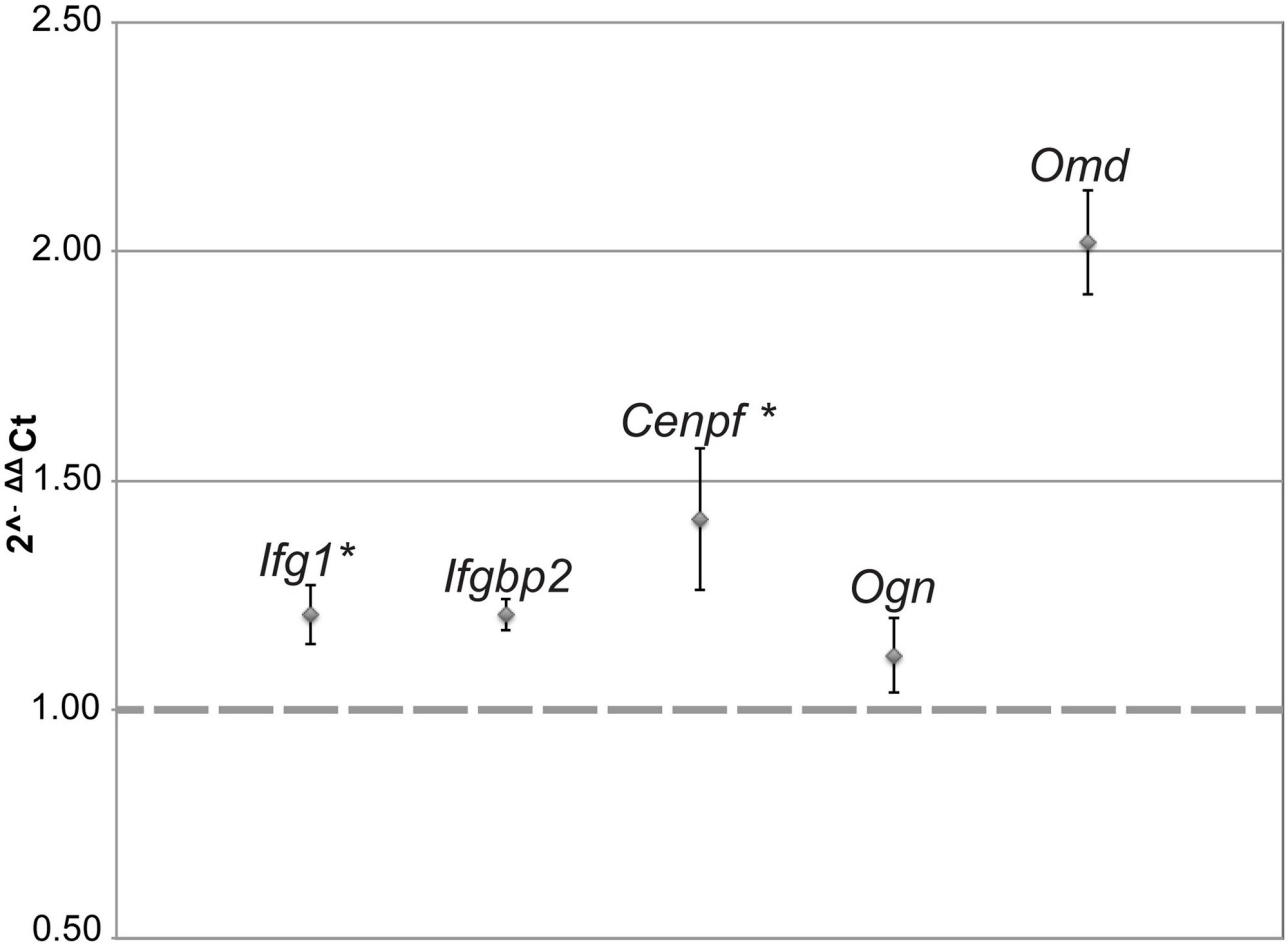
qPCR results. Expression levels of the five genes were normalized using *Actb* levels, which was not differentially expressed in the dataset. The 2^-ΔΔCt^ method was used to calculate the differences and included three replicates of each sample. Those with asterisks are included in Fig 4.

All five transcripts validated with qPCR trended toward upregulation, concordant with RNA-seq results (Fig 3). The only transcript that reached 2-fold change was *Omd*, which codes for a protein that organizes collagen into uniform fibrils, effecting the transmission of force by the ECM [55].

#### Differentially expressed p(adj) ≤ 0.05 and ncRNAs

Table 2 lists highly differentially expressed transcripts (p(adj) ≤ 0.05). *Lars2* is a nuclear-encoded gene whose protein is responsible for charging tRNAs in the mitochondria. No other tRNA synthetases were differentially regulated, ruling out a global downregulation of tRNA function. Inspection of the read profiles using IGV reveals that all reads were restricted to 374 bp of the last exon, indicative of a lncRNA. BLAST analyses of the sequence attributed to the *Lars2* gene against both nucleotide and protein databases revealed no significant homology in other organisms. This is evidence that *Lars2* is a lineage-specific ncRNA [56]. *Gm15564* (245 bp) and *Gm23935* (178 bp) are predicted ncRNAs for which no function is known. *Mir6236* is a microRNA and the reads span 76 bp that map to this validated pre-RNA. *Sorbs2os* is a validated ncRNA although its function is not yet identified.

**Table 2:** Differentially expresses genes (p(adj) ≤ 0.05).

Gene Symbol	Name	Log_2_ fold change	Log CPM	FDR
*Lars2*	leucyl-tRNA synthetase mitochondrial	-1.95	7.15	1.22e-7
*Gm15564*	predicted gene 15564	-2.42	5.41	1.33e-11
*Mir6236*	microRNA 6236	-2.55	4.96	1.77e-8
*Gm23935*	predicted gene 23935	-1.72	4.23	3.60e-4
*Sorbs2os*	sorbin and SH3 domain containing 2 opposite strand	1.51	3.56	1.51e-2
*B3galt2*	UDP-Gal:betaGlcNAc beta 1 3-galactosyltransferase polypeptide 2	2.54	2.88	3.12e-6

*B3galt2* is coded on the opposite strand from the tumor suppressor *Cdc73*, which is also upregulated in this data set. B3galt2 is part of complex localized in the Golgi that catalyzes N-linked glycosylation of lipids and proteins. Targets of N-linked glycosylation include integrins [57] and collagens [58].

Differentially expressed non-coding RNAs (Table 3) include *Neat1* and *Malat1*, which are associated with two ribonucleoprotein complexes in the nucleus, nuclear speckles and paraspeckles, respectively. Both RNAs are known to bind to actively transcribed regions, [59] so upregulation is consistent with rapidly dividing cells. *Ftx* functions as a miRNA sponge during in osteosarcoma cells [22], but its role during normal cell proliferation is not known. There are circRNA annotations associated with the *Ftx* region (a.k.a. B230206F22Rik) in the mouse genome [23, 24], but there is inadequate sequence data in this study to determine if this a linear or a circRNA. The potential miRNAs targets sequestered by *Ftx* are below the cut-off. *Mir17hg* produces miRNAs known to be associated with skeletal development [21].

**Table 3:** Differentially expressed (p≤0.05) non-coding RNAs.

Gene Symbol	Name	Log_2_ FC	Log CPM	p-value
*Malat1*	metastasis associated lung adenocarcinoma transcript 1	0.78	12.8	0.0044
*Neat1*	nuclear paraspeckle assembly transcript 1	1.75	8.2	0.0007
*Ftx*	Ftx transcript Xist (X-inactive specific transcript) regulator	1.07	3.2	0.0048
*Mir17hg*	Mir17 host gene 1	1.08	2.7	0.022

#### Enrichment and network analysis

**Fig 4:**
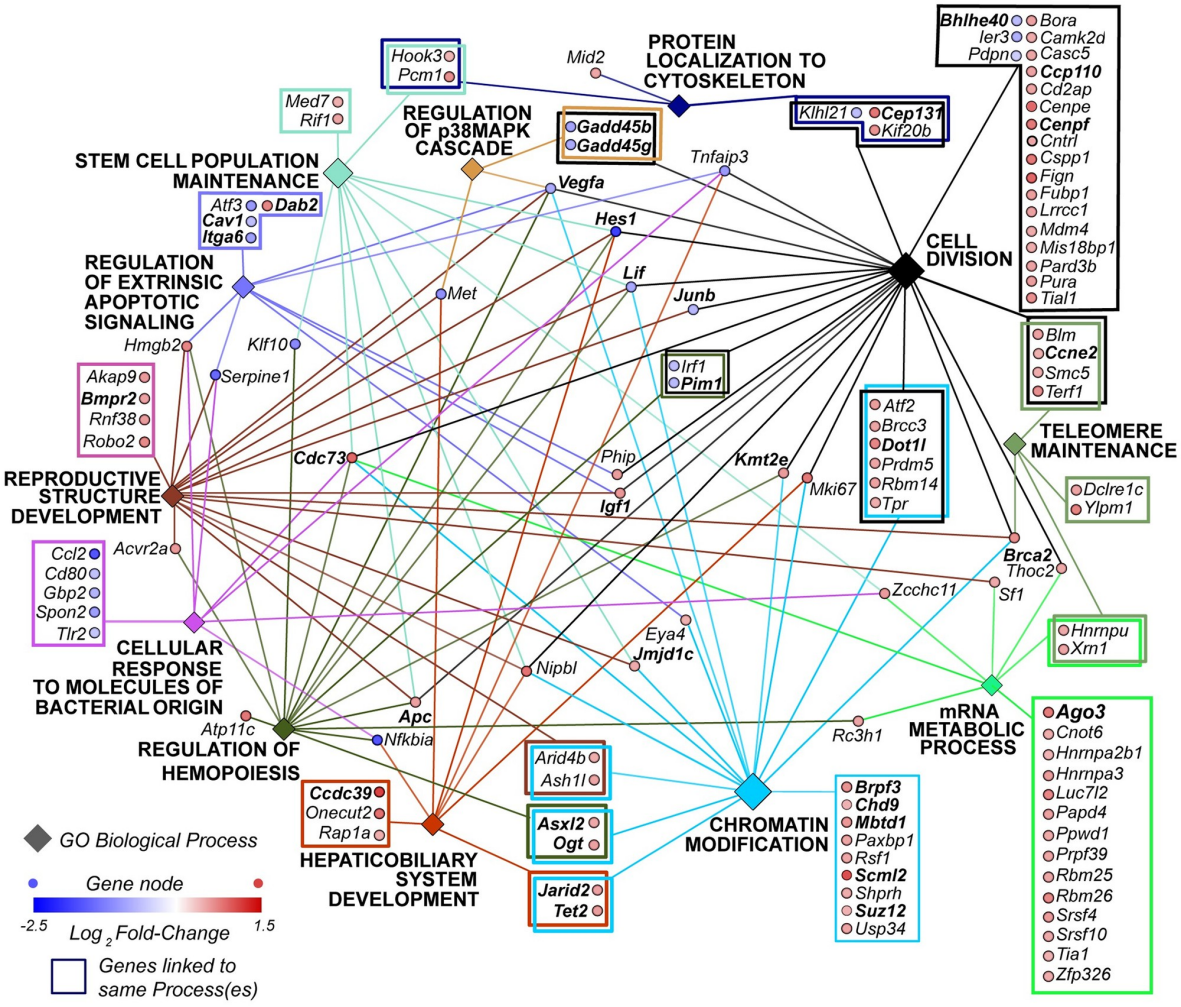
Biological process gene ontology terms enrichment and network analysis. Of the 432 differentially expressed genes with p≤0.05, 117 are retained in this network. Diamond-shaped nodes are GO biological process terms, larger diamonds indicate higher levels of significance, p(adj) value for each GO node is shown. Round nodes are genes, blue nodes are downregulated and red are upregulated. Bolded gene abbreviations in the figure are discussed in the text and full gene names are given in Table 4. S3 Table in S1 File provides this information for all 117 genes.

The 432 differentially expressed (p≤0.05) protein-coding transcripts were subjected to enrichment and network analysis for GO Biological Process terms and 117 transcripts have significant associations (p(adj) ≤ 0.05) to GO terms (Fig 4). GO term enrichment analysis visualized as a network provides a method to assess biological significance that focuses on multiple genes involved in the same processes rather than single gene differential expression. Bar charts (S1 Fig) and pie charts (S2 Fig) are alternative methods of visualization. Some of the GO terms used in Fig 4 are overview terms; these are assigned by ClueGO based upon most significant p(adj) values of closely related terms. Using the overview terms as nodes is a form of framing that improves visualization. In Fig 4, the genes with links to the same processes are framed to further enhance visualization. The cytoscape network as it appears before markup in Illustrator is provided as a .tiff file (S4 Fig. Cytoscape.tiff) and as a cytoscape file (S5 Fig. Cytoscape.cys).

**Table 4:** Network genes discussed in text.

Gene Symbol	Title	Log_2_ FC	log CPM	p-value
*Ago3*	Argonaute RISC catalytic subunit 3	1.0	5.2	0.001
*Apc*	Adenomatosis polyposis coli	0.7	6.1	0.018
*Ash1l*	Ash1 (absent small or homeotic)-like (Drosophila)	0.7	6.7	0.036
*Asxl2*	Additional sex combs like 2 (Drosophila)	0.6	5.3	0.035
*Bhlhe40*	Basic helix-loop-helix family member e40	-0.8	7.2	0.045
*Blm*	Bloom syndrome RecQ helicase-like	0.7	3.6	0.038
*Bmpr2*	Bone morphogenetic protein receptor type II (serine/threonine kinase)	0.7	7.1	0.022
*Brca2*	Breast cancer 2	0.8	5.1	0.005
*Brpf3*	Bromodomain and PHD finger containing 3	0.8	4.0	0.034
*Cav1*	Caveolin protein 1	-0.8	7.4	0.023
*Ccdc39*	Coiled-coil domain containing 39	1.4	2.2	0.001
*Ccne2*	Cyclin E2	0.6	4.9	0.044
*Ccp110*	Centriolar coiled coil protein 110	0.7	4.1	0.028
*Cdc73*	Cell division cycle 73 Paf1/RNA polymerase II complex component	1.1	5.3	0.000
*Cenpf*	Centromere protein F	1.0	7.0	0.001
*Cep131*	Centrosomal protein 131	1.0	2.8	0.007
*Chd9*	Chromodomain helicase DNA binding protein 9	0.6	5.8	0.041
*Dab2*	Disabled 2 mitogen-responsive phosphoprotein	0.9	5.4	0.029
*Dot1l*	DOT1-like histone H3 methyltransferase (S. cerevisiae)	0.9	4.7	0.032
*Gadd45b*	Growth arrest and DNA-damage-inducible 45 beta	-1.1	5.0	0.020
*Gadd45g*	Growth arrest and DNA-damage-inducible 45 gamma	-1.0	4.8	0.015
*Hes1*	Hairy and enhancer of split 1 (Drosophila)	-2.4	6.5	0.001
*Igf1*	Insulin-like growth factor 1	0.8	5.0	0.010
*Itga6*	Integrin alpha 6	-1.2	3.1	0.014
*Jarid2*	Jumonji AT rich interactive domain 2	0.7	3.8	0.048
*Jmjd1c*	Jumonji domain containing 1C	0.6	7.2	0.026
*Junb*	Jun B proto-oncogene	-0.9	8.1	0.020
*Klf10*	Kruppel-like factor 10	-1.5	5.8	0.004
*Kmt2e*	Lysine (K)-specific methyltransferase 2E	0.7	5.9	0.038
*Lif*	Leukemia inhibitory factor	-1.1	3.5	0.014
*Mbtd1*	Mbt domain containing 1	0.8	4.6	0.007
*Met*	Mesenchymal Epithelial Transition proto-oncogene	-1.4	2.2	0.005
*Ogt*	O-linked N-acetylglucosamine (GlcNAc) transferase (UDP-N-acetylglucosamine:polypeptide-N-acetylglucosaminyl transferase)	0.7	6.7	0.016
*Pcm1*	Pericentriolar material 1	0.8	6.6	0.003
*Pim1*	Proviral integration site 1	-0.9	3.6	0.026
*Scml2*	Sex comb on midleg-like 2 (Drosophila)	1.3	2.1	0.001
*Suz12*	Suppressor of zeste 12 homolog (Drosophila)	0.6	6.0	0.036
*Tet2*	Tet methylcytosine dioxygenase 2	0.7	4.4	0.017
*Vegfa*	Vascular endothelial growth factor A	-1.0	5.3	0.019

The observations of improved cell growth on UG-Ti is supported by the upregulation of cell division biological processes. Chromatin regulation processes also dominate the network, an expected outcome because cell division is characterized by rapid changes in chromosome structure. The cell cycle regulator cyclin E2 (*Ccne2*) is also involved in telomere maintenance, another important process in rapidly dividing cells. The processes of protein localization to the cytoskeleton and mRNA metabolism are also upregulated, consistent with the well-established role of the cytoskeleton in mechanosensation and transduction. The upregulated mRNA metabolism gene *Ago3* codes for protein critical to RNA interference (RNAi), a regulatory pathway that modulates the availability of transcripts for translation.

Rapid cell division is not necessarily a healthy condition as it can signal a cancerous state. Transcripts coding for tumor-suppressing proteins (*Apc*, *Brca2*, and *Cdc73*) are upregulated while tumor-promoting protein transcripts are downregulated (*Met*, *Junb*, *and Pim1)*. These results are consistent for cells undergoing controlled division.

GO term enrichment analysis does not take into account experimental design factors, such as cell type and treatment. This type of framing must be applied manually and is guided by the principle that protein isoforms can represent different transcripts produced by the same gene and are dependent upon the cell type, tissue, and temporal factors. Genes are often pleiotropic, affecting different traits. GO term assignments reflect the current state of knowledge and are biased by the status of the GO database. As more gene functions are determined, the results of network analyses will change.

The other processes with more upregulated than downreglated genes include ‘Stem Cell Maintenance,’ ‘Hemopoiesis,’ ‘Hepaticobiliary System Development,’ and ‘Reproductive Structure Development.’ The preosteoblastic state is perpetuated by stem cell maintenance proteins during the osteoconductance stage. Hemopoiesis is justifiable since this process is a function of bone marrow. Differentially regulated primary cilia genes result in the linkage to the hepaticobiliary system because primary cilia are well characterized in liver and bile duct functions [60]. Primary cilia in osteoblasts are known to respond to mechano-stimulation [61]. The linkage of reproductive developmental processes reflects the multiple functions of genes such as *Bmpr2*, which is also expressed in the placenta.

In a network in which upregulated transcripts and processes dominate, it is informative to focus on downregulated entities. Biological processes with more downregulated genes are ‘Cellular Response to Molecules of Bacterial Origin’, ‘Regulation of Extrinsic Apoptotic Signaling’, and ‘Regulation of the p38MAPK Cascade.’ The downregulation of genes that respond to bacteria in cells in contact with UG-Ti suggest that it is less likely to trigger immune system signaling. The downregulation of apoptotic signaling is also indicative of a more cytocompatible substrate. *Dab2* is the sole upregulated gene linked to apoptosis, consistent with its role as an inhibitor of apoptosis. The p38MAPK process known to be under the control of circadian clock and includes two growth arrest genes (*Gadd45b* and *Gadd45g)*, which activate genes by demethylation during stress [62, 63]. Downregulation suggests that the cells are experiencing reduced ‘stress’ on UFG-Ti.

Two of the three downregulated transcription factors that negatively regulate circadian clock genes are members of the basic helix loop helix (Bhlh) family, *Bhlhe40* and *Hes1 (a*.*k*.*a*. *Bhlhe39)*. Hes1 protein has a second function; it binds to and enhances the DNA binding ability of the Runx2 protein [64], another transcription factor that is a well-established effector of osteogenesis. In the present study *Hes1* is downregulated on UFG-Ti; but its effect on Runx2 protein cannot be evaluated with RNAseq data. If the stabilization of Runx2 protein is required for it to function as a transcription factor, *Hes1* downregulation suggests that Runx2’s DNA binding capability is diminished during rapid cell division. The third transcription factor, Klf10, is involved in a variety of cellular processes, including osteoblast differentiation [65]. In the liver, Klf10 connects the circadian rhythm to metabolism [66]. The importance of the circadian clock to cell division and bone metabolism have been established [18, 67], but this complicated relationship has yet to be fully elucidated.

Fig 4 includes 15 genes involved in epigenetics, the biochemical marking of DNA and histones that results in changes in chromatin structure that, in turn, affects transcription rates. The polycomb repressive complexes (PRCs) include PcG proteins that are responsible for marking histones, the protein fraction of chromatin. Upregulation of PcG genes (*Ogt*, *Tet2*, *Suz12*, *Jarid2*, *Asxl2*, *Mbtd1*, *Ash1l*, and *Scml2*) provide evidence for the role of PRCs during osteoconduction. Other transcripts whose products carry out epigenetic functions include *Brpf3*, *Cdc73*, *Dot1l*, *Jmjd1c*, and *Kmt2e*; all are upregulated, as is the chromatin modifier *Chd9*, known to function during osteogenesis [17].

An advantage of viewing enrichment results as a network (Fig 4) rather than a list is that the connections between the processes become apparent. *VegfA* and *Lif* code for cytokines, which have multiple functions in cells depending on the cell type and environment. Both are known to be involved in osteogenesis [15, 16]. The isoform of *VegfA* that is translocated to the nucleus may interact with lamin proteins [12]. PcG proteins also interact with lamin proteins, making both candidate elements that connect the mechanotransduction pathway to osteoconduction.

The role of primary cilia in mechanotransduction is supported by upregulation of genes with functions related to cilia (*Cenpf*, *Ccp110*, *Cep131*, *Pcm1*, *Ccdc39*.) However, this observation may also reflect the established connection between the stage of cell division and primary cilia length [68]. This is another issue that remains to be resolved.

## Discussion

**Fig 5:**
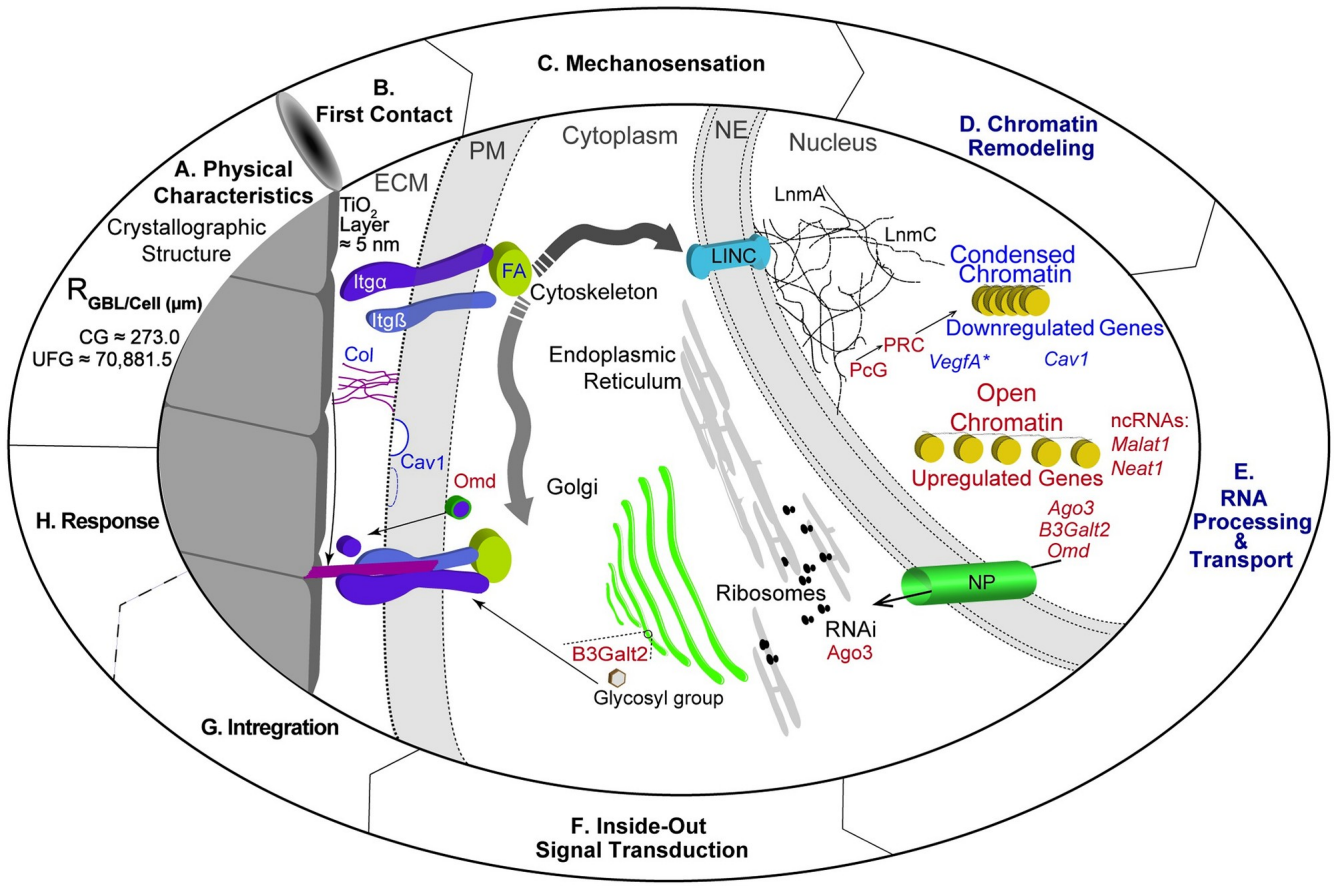
Mechanosensation and transduction model for bio-activating materials. 5A. This model focuses on a single physical characteristic of Ti, the R_GBL/cell_. 5B. First contact involves the extracellular matrix (ECM) domain of integrin α (Itgα) with the substrate, which includes a 5 nm layer of TiO_2_. Contact causes the association with an integrin ß chain (Itg ß), setting off a complex cascade of protein activation in the cytoplasm. 5C. Mechanosensation transmits the force through focal adhesions (FA) and other cytoplasmic protein complexes. Eventually, the signal reaches the linker of nucleoskeleton and cytoskeleton (LINC) complex embedded in the nuclear envelope (NE). 5D. Chromatin remodeling response involves the differential condensation and opening of chromatin. 5E. RNA transcripts are processed depending upon their function and, if required, transported out of the nucleus through nuclear pores (NP). 5F. Inside-out signal transduction includes regulation by the RNA interference (RNAi), transport to a ribosome, passage through the ER (endoplasmic reticulum) and Golgi, any post-translational modifications, and transport to the appropriate compartment. 5G. Integration of the Ti into bone requires the modification of collagen molecules in the ECM in concert with the maturation of FAs. 5H. The response of the substrate and subsequent cell are topics requiring additional research. Not shown in this model is the role of primary cilia.

Ribosome-depleted RNAseq provides a direct measure of ncRNAs. It a recent revelation that ncRNAs are common in genomes, so annotation of these important molecules has lagged behind that of protein coding regions [40]. This limitation is exemplified by the three out of five highly significant (P(adj) ≤ 0.05) differentially expressed genes are annotated ncRNAs with no known function. *Mir17hg* is known to play a role in skeletal processes consistent with a role in during osteoconduction. The evidence for the involvement of circRNAs in osteoconduction is circumstantial due to the current state of databases [69] and the increased coverage necessary to detect these molecules. No piRNAs are detected, but this is likely due to the size selection step. RNA-seq results for validated proteins are consistent with established functions during mechanosensation and transduction, making possible a model of mechanosensation from grain boundaries (Fig 5).

In this model, first contact (Fig 5B), mechanosensation (Fig 5C), and inside-out signaling (Fig 5F) occur as previously determined but are connected through events in the nucleus. Chromatin remodeling (Fig 5D) allows the nucleus to act as a rheostat; the greater the signal coming from the substrate, the more pronounced the condensation and de-condensation of chromosomal domains becomes. RNA processing and transport returns the signal to the cytoplasm (Fig 5E). The model predicts that domains containing genes for division become more open whereas domains that control negative reactions to the substrate, including apoptosis, are more condensed in cells on UG. How grain boundaries influence the mechanosensory process remains to be determined. Our working hypothesis is that protein absorption is positively correlated with surface energy, which in this case is dependent on the density of grain boundaries. There is more energy to which proteins in the media adsorb in UG-Ti, creating a more suitable substrate for cell attachment, mobility, and proliferation. This difference is transduced to the nucleus and results in changes in the 3-dimensional configuration of the nucleus. Once cells become confluent, the chromatin undergoes remodeling that condenses regions necessary for cell division and exposes regions with genes required for osseointegration.

## Conclusions

RNAseq provides a snapshot of the transcriptome after 72 hours on UG-Ti as compared to CG-Ti. Bio-activation is a complex process that involves the regulation of multiple elements of the cytoplasm and the nucleus. Transcriptomic profiles for non-differentially expressed genes supports the established profile for preosteoblasts and quantitates the availability of transcripts for genes involved in mechanosensation, transduction, and osteogenesis. Network analysis data supports hypotheses regarding the involvement of VegfA, Igf1, and Lif proteins in osteogenesis. Further analysis of the interaction of VegfA and PcG with lamin proteins is needed to confirm this connection between mechanosensation and osteogenesis. The role of cilia and caveolae in mechanosensation and the reasons for downregulation of genes and processes under the control of the circadian clock and remain to be determined. While this project focused on the genomic response to grain boundaries in titanium to enhance bio-activating properties, it reveals the potential to understand how materials can bind and inactivate biological material.

The next step is the development of reference substrates with extensively characterized crystallographic and topological features. Parallel biological datasets, including *in situ* protein localization, gene expression, and chromatin remodeling are necessary to validate the model for bio-activation.

## Supporting information

S1 Text Supplemental information. The data presented here includes raw data quantitation, data for genes involved in mechanobiology and osteogenesis, term enrichment visualizations using bar and pie charts, and all gene symbols in Fig 4.
(DOCX)

S1 Fig Visualization of data using a Zipf’s power law graph. The log CPM is plotted against the rank of each gene. All genes in S1 Table in S1 File are shown on the graph as well as protein coding regions with FDR≤0.05 and non-coding RNAs p≤0.05.
(TIF)

S2 Fig Distribution of GO terms associated with differentially express genes (p≤0.05). Only statistically significant associations, p(adj) ≤ 0.05, are shown. The length of each bar is the % of all genes in the genome associated with that GO term. The numbers at the end of each bar are the number of genes differentially regulated associated with the GO term. Overview terms that include multiple GO terms are indicated by vertical lines.
(TIF)

S3 Fig Distribution of overview terms. Font color corresponds to overview term colors in S1 Fig. The numbers in parenthesis indicate the number of GO terms in the overview term, the number of genes in the overview term, and the percentage of the genes that are upregulated.
(TIF)

S4 Fig This is the network in Fig 4 before the use of Illustrator to frame genes linked to the same process(es). (TIF)

S5 Fig This is the cytoscape file (.cys) from which Fig 4 was derived. It can be opened with the public domain software available from https://cytoscape.org/.
(CYS)

S1 File (DOCX)
