# Supplementary figures and images for: Bio-activating ultrafine grain titanium: RNA sequencing reveals enhanced mechano-activation of osteoconduction on nanostructured substrates
Source: PLoS One. 2020 Sep 24;15(9):e0237463. doi: 10.1371/journal.pone.0237463 (PMC7514099; doi:10.1371/journal.pone.0237463)

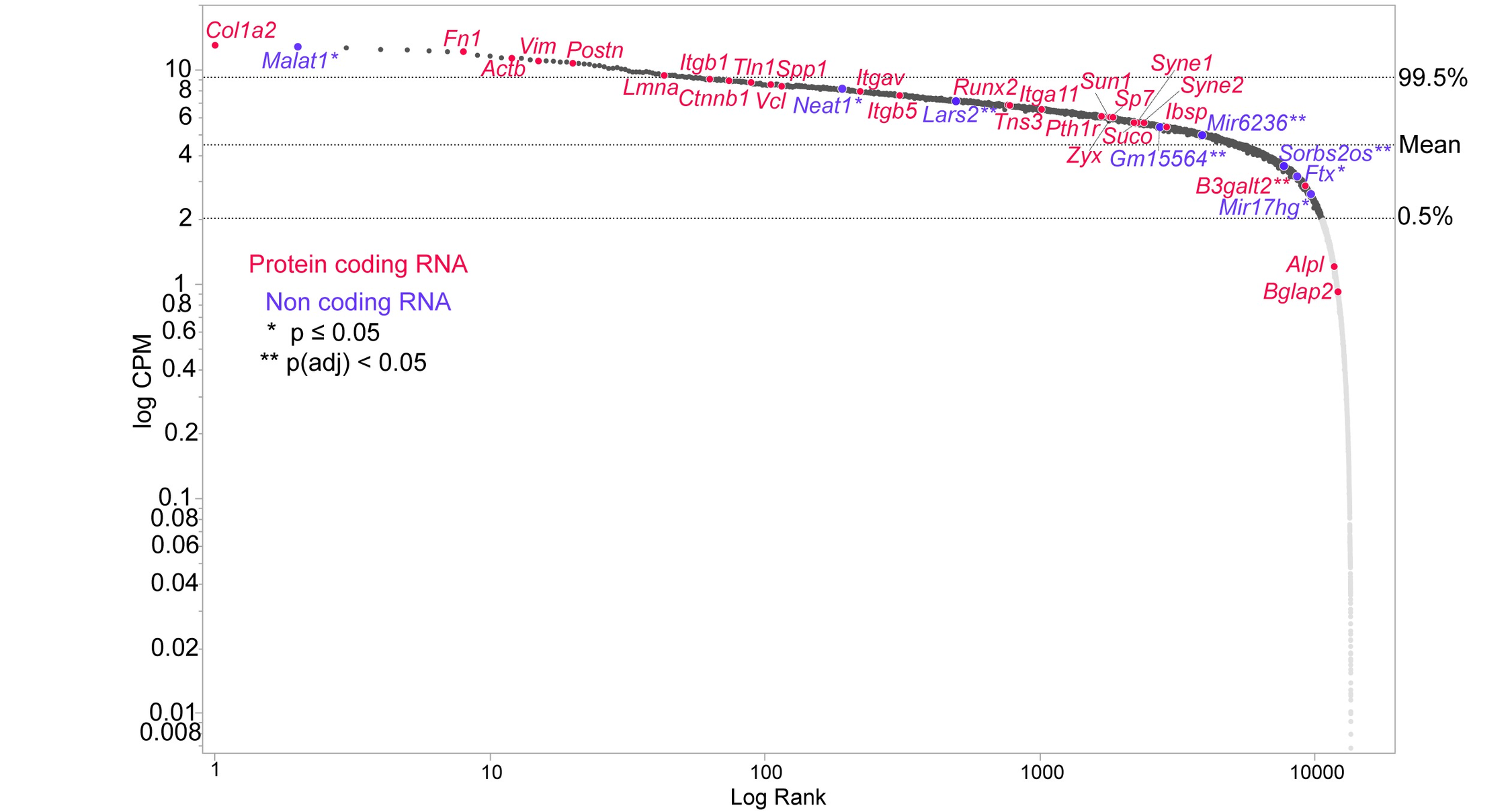

Supplement: S1 Fig — The log CPM is plotted against the rank of each gene. All genes in S1 Table in S1 File are shown on the graph as well as protein coding regions with FDR≤0.05 and non-coding RNAs p≤0.05. (TIF) [file pone.0237463.s002.tif]

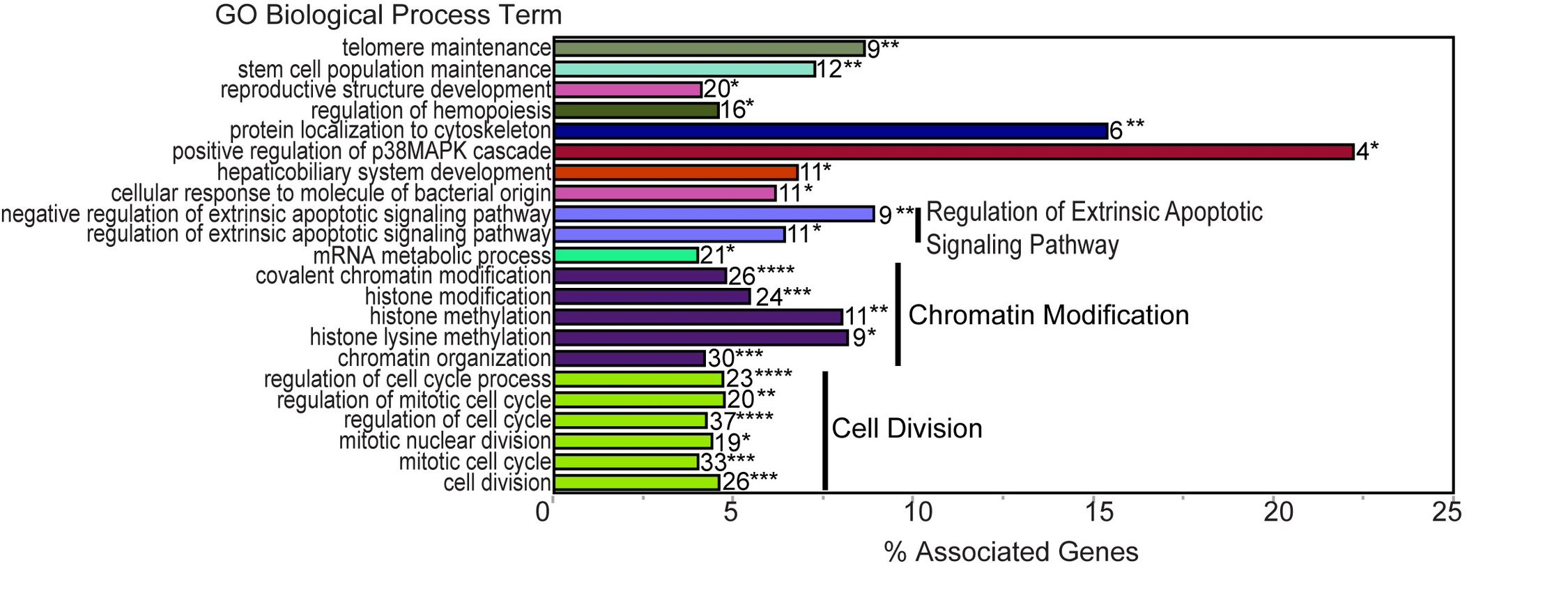

Supplement: S2 Fig — Only statistically significant associations, p(adj) ≤ 0.05, are shown. The length of each bar is the % of all genes in the genome associated with that GO term. The numbers at the end of each bar are the number of genes differentially regulated associated with the GO term. Overview terms that include multiple GO terms are indicated by vertical lines. (TIF) [file pone.0237463.s003.tif]

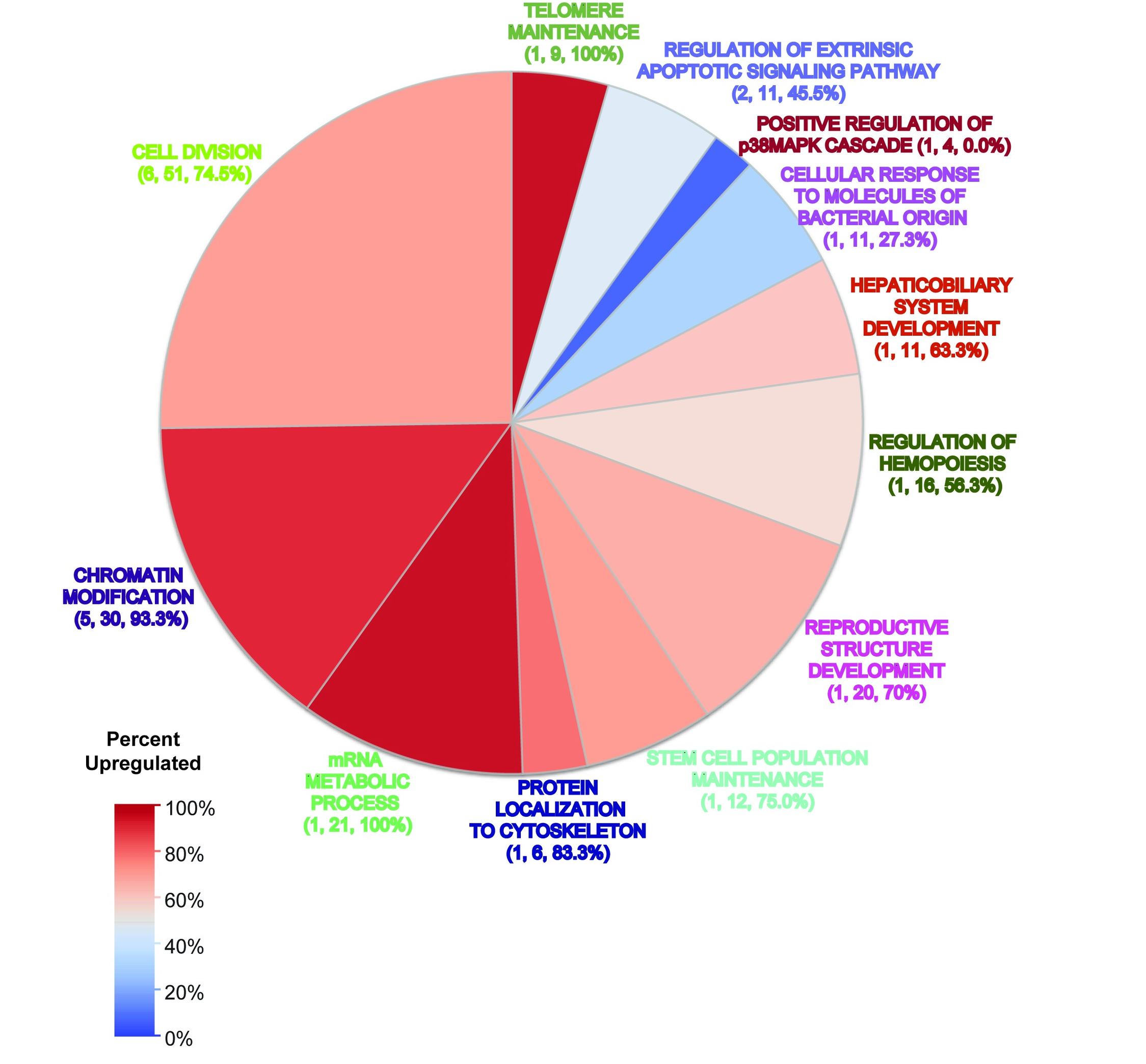

Supplement: S3 Fig — Font color corresponds to overview term colors in S1 Fig. The numbers in parenthesis indicate the number of GO terms in the overview term, the number of genes in the overview term, and the percentage of the genes that are upregulated. (TIF) [file pone.0237463.s004.tif]

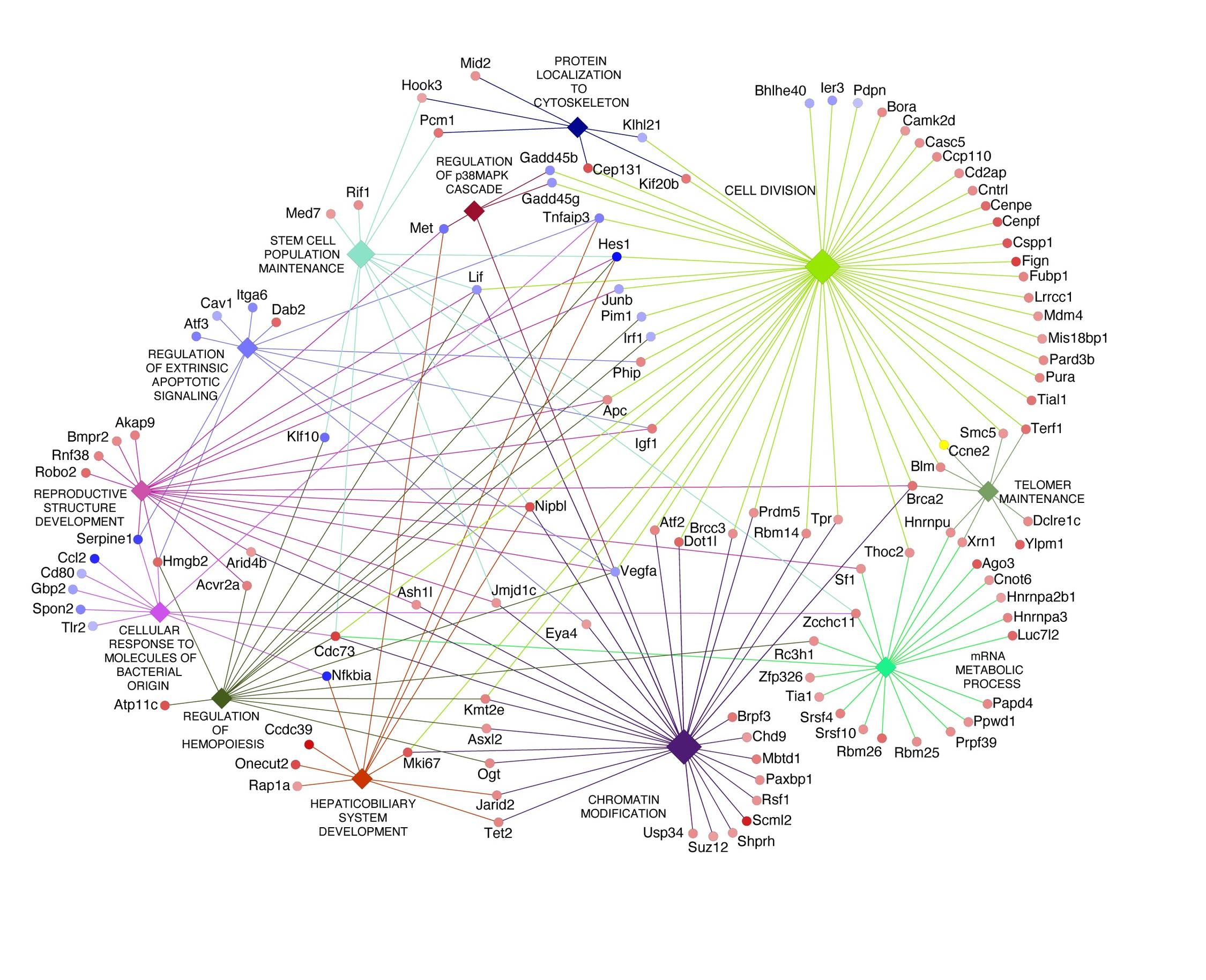

Supplement: S4 Fig — (TIF) [file pone.0237463.s005.tif]
