# Supplementary material for: Bio-activating ultrafine grain titanium: RNA sequencing reveals enhanced mechano-activation of osteoconduction on nanostructured substrates
Source: PLoS One. 2020 Sep 24;15(9):e0237463. doi: 10.1371/journal.pone.0237463 (PMC7514099; doi:10.1371/journal.pone.0237463)
Supplement: S1 File — (DOCX) [file pone.0237463.s007.docx]

Bio-activating ultrafine grain titanium: RNA sequencing reveals enhanced mechano-activation of osteoconduction on nanostructured substrates

Rebecca A. Reiss^1^ *¶ , Terry C. Lowe^2^ ¶ , Johnny A. Sena^3&^, Oleg Makhnin^4&^, Melanie C. Connick^1&^, Patrick E. Illescas^1&^, Casey F. Davis^2&^

^1^ Biology Department, New Mexico Institution of Mining and Technology, Socorro, NM, 87801, USA

^2^ George S. Ansell Department of Metallurgical and Materials Engineering, Colorado School of Mines, Golden, CO 80401, USA

^3^ National Center for Genome Resources, Santa Fe, NM, 87505, USA

^4^ Mathematics Department, New Mexico Institute of Mining and Technology, Socorro, NM, 87801, USA

* Corresponding author

E-mail: rebecca.reiss@nmt.edu

¶These authors contributed equally to this work.

&These authors also contributed equally to this work.

**Supplemental Information**

**Raw Data**

The data from Illumina sequencing and read quality control are provided in Table S1. CG2, CG3, UG4, and UG5 are biological replicates that were grown in the same cell culture plate. The 48,969,121 unique reads define over 20,000 genes.

**Table S1. Raw data results**

| **Sample** | **Reads passing Samtools Flagstat QC** | **Reads mapping to reference** | **Percent unique reads** |
| --- | --- | --- | --- |
| CG2 | 11,398,784 | 9,253,164 | 81.2 |
| CG3 | 16,383,125 | 13,974,378 | 85.3 |
| UG4 | 16,358,870 | 13,698,966 | 83.7 |
| UG5 | 14,981,590 | 12,042,613 | 80.4 |
| **Total** | **59,122,369** | **48,969,121** | **82.65** |

**Genes of interest in mechanosensation, transduction and osteogenesis**

Table S2 is the data for a list of genes whose products are involved in mechanosensation, transduction, and osteogenesis. These are not differentially expressed. The table is sorted by log CPM, indicating the number reads that map each annotation and is used here as a proxy for the abundance of transcripts. The mean CPM is 4.4 and any annotations less than 2.0 were filtered from the gene list. The table includes annotations with log CPMs below 2, because of their importance later in osteogenesis.

Of the over 20,000 transcripts evaluated, collagen type I alpha 2 (*Col1a2*) is the most abundant (CPM 13.03). The extracellular matrix (ECM) is the first part of the cell to encounter a substrate and includes Col1a2, fibronectin 1 (Fn1), and integrins. Integrin functions are well established in mechanotransduction and the highest expressed are *ItgaV*, *Itgba11*, *Itgb1*, and *Itgb5*, and *Itgba11* (Table 2). Dimers of αV/ β1 and αV/ β5 are known to bind the ligand tripeptide motif Arginine-Glycine-Aspargine (RGD), while α11/ β1 binds collagen. The binding properties of α11/ β5 have yet to be established.

Once a signal is transduced through the membrane into the cytoplasm by integrins, it moves through the cytoskeleton, which involves a complex series of interactions with multiple proteins. Table S2 and Figure S1 include cytoskeleton-coding genes that are abundant in the RNAseq data set, including transcripts for focal adhesion proteins actin (*Actb*), tensin 3 (*Tns3*), fibronectin (*Fn1*), vinculin (*Vcl*), zyxin (*Zyx*), and talin (*Tln1*). Vimentin (*Vim*) is a type III intermediate filament used a marker for mesenchymal cells. Beta-catenin (*Ctnnb1*) transcripts code for an abundant protein that functions in cell adhesion and also transduces signal via the Wnt signaling pathway.

After transmission through proteins of the cytoskeleton, the signal reaches the linker of the nucleoskeleton and cytoskeleton (LINC) complex, located in the nuclear envelope, which modulates genomic-wide transcription patterns in response to substrate stiffness. The LINC complex is coded for by spectrin repeat containing nuclear envelope 1 and 2 genes (*Syne1* and *Syne2*), and the SUN domain containing ossification factor (*Sun1*) gene. The LINC complex interacts with lamin A (*Lnma*) gene products that line the interior of the nuclear envelope and are involved in the 3 -dimensional structure of the nucleus.

Transcripts that produce proteins with known functions during osteogenesis that are expressed after 72 hours include secreted phosphoprotein 1 (*Ssp1*), Runt related transcription factor 2 (*RunX2*), Sp7 transcription factor 7 (*Sp7*), and integrin binding sialoprotein (*Ibsp*). Alkaline phosphatase liver/bone/kidney (*Alpl*) and bone gamma-carboxyglutamate protein 2 (*Bglap2*) are expressed, but at a level below the log 2 CPM cutoff. Transcripts for Bone morphogenetic proteins 2 and 7 (*Bmp2* and *Bmp7*) are not detected.

**Table S2. Data for genes of interest in mechanotransduction and osteogenesis**

| **Gene Symbol** | **Title** | **Alias(es)** | **log CPM^1^** |
| --- | --- | --- | --- |
| *Col1a2* | collagen type I alpha 2 | α2(I) | 13.03 |
| *Fn1* | fibronectin 1 |  | 12.17 |
| *Actb* | actin beta |  | 11.34 |
| *Vim* | vimentin |  | 11.01 |
| *Postn* | periostin osteoblast specific factor | Osf-2 | 10.75 |
| *Lmna* | lamin A |  | 9.43 |
| *Itgb1* | integrin beta 1 (fibronectin receptor beta) | β1 | 9.06 |
| *Ctnnb1* | catenin (cadherin associated protein) beta 1 |  | 8.91 |
| *Tln1* | talin 1 |  | 8.74 |
| *Vcl* | vinculin |  | 8.55 |
| *Spp1* | secreted phosphoprotein 1 | Opn, Osteopontin,  bone sialoprotein I | 8.39 |
| *Itgav* | integrin alpha V | αV | 7.95 |
| *Itgb5* | integrin beta 5 | β5 | 7.60 |
| *Runx2* | runt related transcription factor 2 | Osf Transcription factor | 6.87 |
| *Tns3* | tensin 3 |  | 6.83 |
| *Itga11* | integrin alpha 11 | α11 | 6.55 |
| *Pth1r* | parathyroid hormone 1 receptor | Pthr | 6.08 |
| *Sun1* | Sad1 and UNC84 domain containing 1 |  | 6.04 |
| *Zyx* | zyxin |  | 6.03 |
| *Sp7* | Sp7 transcription factor 7 | *Osx*, Osterix | 6.01 |
| *Suco* | SUN domain containing ossification factor | Osteopotentia | 5.68 |
| *Syne1* | spectrin repeat containing nuclear envelope 1 | Nesprin 1 | 5.67 |
| *Syne2* | spectrin repeat containing nuclear envelope 2 | Nesprin 2 | 5.67 |
| *Ibsp* | integrin binding sialoprotein | Bsp, bone sialoprotein | 5.42 |
| *Alpl* | alkaline phosphatase liver/bone/kidney | Alp | 1.21 |
| *Bglap2* | bone gamma-carboxyglutamate protein 2 | Ocn, Osteocalcin | -0.90 |
| *Bmp2* | bone morphogenetic protein 2 |  | ND^2^ |
| *Bmp7* | bone morphogenetic protein 7 |  | ND |

1. The location of these genes in relationship to the data distribution are shown in red (Figure S1)

2. ND No reads detected

**S1 Fig S1. Visualization of data using a Zipf’s power law graph**. The log CPM is plotted against the rank of each gene**.**  All genes in Table S1 are shown on the graph as well as protein coding regions with FDR≤0.05 and non-coding RNAs p≤0.05.

**Term Enrichment**

**Cytoscape/ClueGO parameters**

Number of genes with p ≤0.05 uploaded to cytoscape-432.

Genes with GO terms - 117.

GO levels 3 – 8

Minimum numbers of genes per term - 3

Minimum percentage of genes - 4.0%

GO term fusion ON

GO group – ON

Overview term smallest - P value

Statistical Test Used = Enrichment/Depletion (Two-sided hypergeometric test)

Correction Method Used = Bonferroni step down

Kappa score threshold 0.4.

The distribution of genes to the 12 GO overview process terms is shown in Figure 2. The percent of upregulated genes in each process is shown by color. Genes involved in cell division, telomere maintenance, and RNA metabolism are predominately up-regulated, while apoptosis and cellular response to bacteria are downregulated.

GO terms can be categorized by overview terms that describe a process common to related groups of biological processes. The data are associated to 22 GO terms that are assigned to 12 overview terms (Figure S2). Cell division is represented by six GO terms, chromatin modification by five terms, and regulation of extrinsic apoptosis by two. The rest of the overview terms are associated with a single GO term. The distribution of data annotated with GO overview terms is shown as a pie chart in Figure S3.

**S1 Fig S2. Distribution of GO terms associated with differentially express genes (p≤0.05).** Only statistically significant associations, p(adj) ≤ 0.05, are shown. The length of each bar is the % of all genes in the genome associated with that GO term. The numbers at the end of each bar are the number of genes differentially regulated associated with the GO term. Overview terms that include multiple GO terms are indicated by vertical lines.

**S1 Figure S3.** Distribution of overview terms. Font color corresponds to overview colors in Figure S1. The numbers in parenthesis indicate the number of GO terms in the overview term, the number of genes in the overview term, and the percentage of the genes that are upregulated.

**Table S3 Key to Fig 4 gene symbols.** Title includes annotation source, CPM=Counts per million, FC=fold change. Entries in bold are discussed in the text.

| **Gene Symbol** | **Title** | **Log_10_ FC** | **Log_10_ CPM** | **p-value** |
| --- | --- | --- | --- | --- |
| *Acvr2a* | activin receptor IIA [Source:MGI Symbol;Acc:MGI:102806] | 0.75 | 4.95 | 0.009 |
| ***Ago3*** | **argonaute RISC catalytic subunit 3 [Source:MGI Symbol;Acc:MGI:2446634]** | **0.98** | **5.22** | **0.001** |
| *Akap9* | A kinase (PRKA) anchor protein (yotiao) 9 [Source:MGI Symbol;Acc:MGI:2178217] | 0.75 | 6.39 | 0.012 |
| ***Apc*** | **adenomatosis polyposis coli [Source:MGI Symbol;Acc:MGI:88039]** | **0.70** | **6.06** | **0.018** |
| *Arid4b* | AT rich interactive domain 4B (RBP1-like) [Source:MGI Symbol;Acc:MGI:2137512] | 0.60 | 5.22 | 0.049 |
| ***Ash1l*** | **ash1 (absent small or homeotic)-like (Drosophila) [Source:MGI Symbol;Acc:MGI:2183158]** | **0.66** | **6.67** | **0.036** |
| ***Asxl2*** | **additional sex combs like 2 (Drosophila) [Source:MGI Symbol;Acc:MGI:1922552]** | **0.64** | **5.27** | **0.035** |
| *Atf2* | activating transcription factor 2 [Source:MGI Symbol;Acc:MGI:109349] | 0.73 | 5.26 | 0.010 |
| *Atf3* | activating transcription factor 3 [Source:MGI Symbol;Acc:MGI:109384] | -1.27 | 4.00 | 0.002 |
| *Atp11c* | ATPase class VI type 11C [Source:MGI Symbol;Acc:MGI:1859661] | 1.02 | 3.99 | 0.001 |
| ***Bhlhe40*** | **basic helix-loop-helix family member e40 [Source:MGI Symbol;Acc:MGI:1097714]** | **-0.84** | **7.25** | **0.045** |
| ***Blm*** | **Bloom syndrome RecQ helicase-like [Source:MGI Symbol;Acc:MGI:1328362]** | **0.69** | **3.63** | **0.038** |
| ***Bmpr2*** | **bone morphogenetic protein receptor type II (serine/threonine kinase) [Source:MGI Symbol;Acc:MGI:1095407]** | **0.70** | **7.14** | **0.022** |
| *Bora* | bora aurora kinase A activator [Source:MGI Symbol;Acc:MGI:1924994] | 0.69 | 3.49 | 0.043 |
| ***Brca2*** | **breast cancer 2 [Source:MGI Symbol;Acc:MGI:109337]** | **0.83** | **5.09** | **0.005** |
| *Brcc3* | BRCA1/BRCA2-containing complex subunit 3 [Source:MGI Symbol;Acc:MGI:2389572] | 0.67 | 4.14 | 0.039 |
| ***Brpf3*** | **bromodomain and PHD finger containing 3 [Source:MGI Symbol;Acc:MGI:2146836]** | **0.81** | **3.96** | **0.034** |
| *Camk2d* | calcium/calmodulin-dependent protein kinase II delta [Source:MGI Symbol;Acc:MGI:1341265] | 0.61 | 6.16 | 0.029 |
| *Casc5* | cancer susceptibility candidate 5 [Source:MGI Symbol;Acc:MGI:1923714] | 0.66 | 5.79 | 0.023 |
| ***Cav1*** | **caveolin 1 caveolae protein [Source:MGI Symbol;Acc:MGI:102709]** | **-0.83** | **7.37** | **0.023** |
| ***Ccdc39*** | **coiled-coil domain containing 39 [Source:MGI Symbol;Acc:MGI:1289263]** | **1.41** | **2.23** | **0.001** |
| *Ccl2* | chemokine (C-C motif) ligand 2 [Source:MGI Symbol;Acc:MGI:98259] | -2.10 | 3.35 | 0.007 |
| ***Ccne2*** | **cyclin E2 [Source:MGI Symbol;Acc:MGI:1329034]** | **0.59** | **4.93** | **0.044** |
| ***Ccp110*** | **centriolar coiled coil protein 110 [Source:MGI Symbol;Acc:MGI:2141942]** | **0.69** | **4.14** | **0.028** |
| *Cd2ap* | CD2-associated protein [Source:MGI Symbol;Acc:MGI:1330281] | 0.67 | 5.64 | 0.017 |
| *Cd80* | CD80 antigen [Source:MGI Symbol;Acc:MGI:101775] | -0.77 | 3.04 | 0.034 |
| ***Cdc73*** | **cell division cycle 73 Paf1/RNA polymerase II complex component [Source:MGI Symbol;Acc:MGI:2384876]** | **1.12** | **5.30** | **0.000** |
| *Cenpe* | centromere protein E [Source:MGI Symbol;Acc:MGI:1098230] | 0.89 | 5.93 | 0.008 |
| ***Cenpf*** | **centromere protein F [Source:MGI Symbol;Acc:MGI:1313302]** | **0.99** | **6.99** | **0.001** |
| ***Cep131*** | **centrosomal protein 131 [Source:MGI Symbol;Acc:MGI:107440]** | **1.02** | **2.82** | **0.007** |
| ***Chd9*** | **chromodomain helicase DNA binding protein 9 [Source:MGI Symbol;Acc:MGI:1924001]** | **0.58** | **5.75** | **0.041** |
| *Cnot6* | CCR4-NOT transcription complex subunit 6 [Source:MGI Symbol;Acc:MGI:2144529] | 0.62 | 6.64 | 0.049 |
| *Cntrl* | centriolin [Source:MGI Symbol;Acc:MGI:1889576] | 0.68 | 5.36 | 0.015 |
| *Cspp1* | centrosome and spindle pole associated protein 1 [Source:MGI Symbol;Acc:MGI:2681832] | 0.99 | 4.66 | 0.001 |
| ***Dab2*** | **disabled 2 mitogen-responsive phosphoprotein [Source:MGI Symbol;Acc:MGI:109175]** | **0.89** | **5.38** | **0.029** |
| *Dclre1c* | DNA cross-link repair 1C PSO2 homolog (S. cerevisiae) [Source:MGI Symbol;Acc:MGI:2441769] | 0.68 | 3.21 | 0.037 |
| ***Dot1l*** | **DOT1-like histone H3 methyltransferase (S. cerevisiae) [Source:MGI Symbol;Acc:MGI:2143886]** | **0.90** | **4.71** | **0.032** |
| *Eya4* | eyes absent 4 homolog (Drosophila) [Source:MGI Symbol;Acc:MGI:1337104] | 0.59 | 6.20 | 0.038 |
| *Fign* | fidgetin [Source:MGI Symbol;Acc:MGI:1890647] | 1.13 | 2.43 | 0.020 |
| *Fubp1* | far upstream element (FUSE) binding protein 1 [Source:MGI Symbol;Acc:MGI:1196294] | 0.73 | 6.08 | 0.009 |
| ***Gadd45b*** | **growth arrest and DNA-damage-inducible 45 beta [Source:MGI Symbol;Acc:MGI:107776]** | **-1.12** | **4.98** | **0.020** |
| ***Gadd45g*** | **growth arrest and DNA-damage-inducible 45 gamma [Source:MGI Symbol;Acc:MGI:1346325]** | **-1.03** | **4.85** | **0.015** |
| *Gbp2* | guanylate binding protein 2 [Source:MGI Symbol;Acc:MGI:102772] | -0.94 | 3.12 | 0.012 |
| ***Hes1*** | **hairy and enhancer of split 1 (Drosophila) [Source:MGI Symbol;Acc:MGI:104853]** | **-2.38** | **6.53** | **0.001** |
| *Hmgb2* | high mobility group box 2 [Source:MGI Symbol;Acc:MGI:96157] | 0.90 | 2.82 | 0.014 |
| *Hnrnpa2b1* | heterogeneous nuclear ribonucleoprotein A2/B1 [Source:MGI Symbol;Acc:MGI:104819] | 0.57 | 8.13 | 0.046 |
| *Hnrnpa3* | heterogeneous nuclear ribonucleoprotein A3 [Source:MGI Symbol;Acc:MGI:1917171] | 0.73 | 3.97 | 0.033 |
| *Hnrnpu* | heterogeneous nuclear ribonucleoprotein U [Source:MGI Symbol;Acc:MGI:1858195] | 0.66 | 6.99 | 0.022 |
| *Hook3* | hook homolog 3 (Drosophila) [Source:MGI Symbol;Acc:MGI:2443554] | 0.55 | 6.67 | 0.046 |
| *Ier3* | immediate early response 3 [Source:MGI Symbol;Acc:MGI:104814] | -0.98 | 6.06 | 0.032 |
| ***Igf1*** | **insulin-like growth factor 1 [Source:MGI Symbol;Acc:MGI:96432]** | **0.78** | **4.98** | **0.010** |
| *Irf1* | interferon regulatory factor 1 [Source:MGI Symbol;Acc:MGI:96590] | -0.80 | 5.61 | 0.034 |
| ***Itga6*** | **integrin alpha 6 [Source:MGI Symbol;Acc:MGI:96605]** | **-1.18** | **3.05** | **0.014** |
| ***Jarid2*** | **jumonji AT rich interactive domain 2 [Source:MGI Symbol;Acc:MGI:104813]** | **0.70** | **3.78** | **0.048** |
| ***Jmjd1c*** | **jumonji domain containing 1C [Source:MGI Symbol;Acc:MGI:1918614]** | **0.62** | **7.18** | **0.026** |
| ***Junb*** | **jun B proto-oncogene [Source:MGI Symbol;Acc:MGI:96647]** | **-0.89** | **8.11** | **0.020** |
| *Kif20b* | kinesin family member 20B [Source:MGI Symbol;Acc:MGI:2444576] | 0.80 | 5.68 | 0.017 |
| ***Klf10*** | **Kruppel-like factor 10 [Source:MGI Symbol;Acc:MGI:1101353]** | **-1.45** | **5.78** | **0.004** |
| *Klhl21* | kelch-like 21 [Source:MGI Symbol;Acc:MGI:1919288] | -0.81 | 3.94 | 0.019 |
| ***Kmt2e*** | **lysine (K)-specific methyltransferase 2E [Source:MGI Symbol;Acc:MGI:1924825]** | **0.74** | **5.89** | **0.038** |
| ***Lif*** | **leukemia inhibitory factor [Source:MGI Symbol;Acc:MGI:96787]** | **-1.08** | **3.53** | **0.014** |
| *Lrrcc1* | leucine rich repeat and coiled-coil domain containing 1 [Source:MGI Symbol;Acc:MGI:1918960] | 0.70 | 4.77 | 0.018 |
| *Luc7l2* | LUC7-like 2 (S. cerevisiae) [Source:MGI Symbol;Acc:MGI:2183260] | 0.90 | 7.71 | 0.001 |
| ***Mbtd1*** | **mbt domain containing 1 [Source:MGI Symbol;Acc:MGI:2143977]** | **0.77** | **4.62** | **0.007** |
| *Mdm4* | transformed mouse 3T3 cell double minute 4 [Source:MGI Symbol;Acc:MGI:107934] | 0.62 | 7.03 | 0.030 |
| *Med7* | mediator complex subunit 7 [Source:MGI Symbol;Acc:MGI:1913463] | 0.59 | 4.11 | 0.043 |
| ***Met*** | **met proto-oncogene [Source:MGI Symbol;Acc:MGI:96969]** | **-1.39** | **2.16** | **0.005** |
| *Mid2* | midline 2 [Source:MGI Symbol;Acc:MGI:1344333] | 0.66 | 4.98 | 0.026 |
| *Mis18bp1* | MIS18 binding protein 1 [Source:MGI Symbol;Acc:MGI:2145099] | 0.61 | 4.20 | 0.048 |
| *Mki67* | antigen identified by monoclonal antibody Ki 67 [Source:MGI Symbol;Acc:MGI:106035] | 0.99 | 8.53 | 0.000 |
| *Nfkbia* | nuclear factor of kappa light polypeptide gene enhancer in B cells inhibitor alpha [Source:MGI Symbol;Acc:MGI:104741] | -2.12 | 5.19 | 0.001 |
| *Nipbl* | Nipped-B homolog (Drosophila) [Source:MGI Symbol;Acc:MGI:1913976] | 1.04 | 6.42 | 0.000 |
| ***Ogt*** | **O-linked N-acetylglucosamine (GlcNAc) transferase (UDP-N-acetylglucosamine:polypeptide-N-acetylglucosaminyl transferase) [Source:MGI Symbol;Acc:MGI:1339639]** | **0.70** | **6.66** | **0.016** |
| *Onecut2* | one cut domain family member 2 [Source:MGI Symbol;Acc:MGI:1891408] | 1.06 | 3.85 | 0.023 |
| *Papd4* | PAP associated domain containing 4 [Source:MGI Symbol;Acc:MGI:2140950] | 0.70 | 2.74 | 0.048 |
| *Pard3b* | par-3 family cell polarity regulator beta [Source:MGI Symbol;Acc:MGI:1919301] | 0.69 | 3.66 | 0.044 |
| *Paxbp1* | PAX3 and PAX7 binding protein 1 [Source:MGI Symbol;Acc:MGI:1914617] | 0.66 | 5.43 | 0.027 |
| ***Pcm1*** | **pericentriolar material 1 [Source:MGI Symbol;Acc:MGI:1277958]** | **0.82** | **6.65** | **0.003** |
| *Pdpn* | podoplanin [Source:MGI Symbol;Acc:MGI:103098] | -0.61 | 5.70 | 0.049 |
| *Phip* | pleckstrin homology domain interacting protein [Source:MGI Symbol;Acc:MGI:1932404] | 0.72 | 6.64 | 0.018 |
| ***Pim1*** | **proviral integration site 1 [Source:MGI Symbol;Acc:MGI:97584]** | **-0.86** | **3.60** | **0.026** |
| *Ppwd1* | peptidylprolyl isomerase domain and WD repeat containing 1 [Source:MGI Symbol;Acc:MGI:2443069] | 0.65 | 3.35 | 0.049 |
| *Prdm5* | PR domain containing 5 [Source:MGI Symbol;Acc:MGI:1918029] | 0.66 | 5.63 | 0.022 |
| *Prpf39* | PRP39 pre-mRNA processing factor 39 homolog (yeast) [Source:MGI Symbol;Acc:MGI:104602] | 0.68 | 5.41 | 0.020 |
| *Pura* | purine rich element binding protein A [Source:MGI Symbol;Acc:MGI:103079] | 0.73 | 5.53 | 0.009 |
| *Rap1a* | RAS-related protein-1a [Source:MGI Symbol;Acc:MGI:97852] | 0.60 | 3.72 | 0.048 |
| *Rbm14* | RNA binding motif protein 14 [Source:MGI Symbol;Acc:MGI:1929092] | 0.69 | 4.69 | 0.028 |
| *Rbm25* | RNA binding motif protein 25 [Source:MGI Symbol;Acc:MGI:1914289] | 0.67 | 6.65 | 0.028 |
| *Rbm26* | RNA binding motif protein 26 [Source:MGI Symbol;Acc:MGI:1921463] | 0.88 | 5.40 | 0.002 |
| *Rc3h1* | RING CCCH (C3H) domains 1 [Source:MGI Symbol;Acc:MGI:2685397] | 0.66 | 6.02 | 0.020 |
| *Rif1* | Rap1 interacting factor 1 homolog (yeast) [Source:MGI Symbol;Acc:MGI:1098622] | 0.67 | 6.37 | 0.024 |
| *Rnf38* | ring finger protein 38 [Source:MGI Symbol;Acc:MGI:1920719] | 0.74 | 5.30 | 0.031 |
| *Robo2* | roundabout homolog 2 (Drosophila) [Source:MGI Symbol;Acc:MGI:1890110] | 0.87 | 5.70 | 0.020 |
| *Rsf1* | remodeling and spacing factor 1 [Source:MGI Symbol;Acc:MGI:2682305] | 0.66 | 5.27 | 0.021 |
| ***Scml2*** | **sex comb on midleg-like 2 (Drosophila) [Source:MGI Symbol;Acc:MGI:1340042]** | **1.32** | **2.10** | **0.001** |
| *Serpine1* | serine (or cysteine) peptidase inhibitor clade E member 1 [Source:MGI Symbol;Acc:MGI:97608] | -1.86 | 6.42 | 0.002 |
| *Sf1* | splicing factor 1 [Source:MGI Symbol;Acc:MGI:1095403] | 0.64 | 6.32 | 0.049 |
| *Shprh* | SNF2 histone linker PHD RING helicase [Source:MGI Symbol;Acc:MGI:1917581] | 0.61 | 5.60 | 0.034 |
| *Smc5* | structural maintenance of chromosomes 5 [Source:MGI Symbol;Acc:MGI:2385088] | 0.62 | 6.13 | 0.032 |
| *Spon2* | spondin 2 extracellular matrix protein [Source:MGI Symbol;Acc:MGI:1923724] | -1.21 | 3.16 | 0.006 |
| *Srsf10* | serine/arginine-rich splicing factor 10 [Source:MGI Symbol;Acc:MGI:1333805] | 0.64 | 5.95 | 0.029 |
| *Srsf4* | serine/arginine-rich splicing factor 4 [Source:MGI Symbol;Acc:MGI:1890577] | 0.75 | 4.09 | 0.039 |
| ***Suz12*** | **suppressor of zeste 12 homolog (Drosophila) [Source:MGI Symbol;Acc:MGI:1261758]** | **0.59** | **6.03** | **0.036** |
| *Terf1* | telomeric repeat binding factor 1 [Source:MGI Symbol;Acc:MGI:109634] | 0.84 | 3.04 | 0.018 |
| ***Tet2*** | **tet methylcytosine dioxygenase 2 [Source:MGI Symbol;Acc:MGI:2443298]** | **0.73** | **4.38** | **0.017** |
| *Thoc2* | THO complex 2 [Source:MGI Symbol;Acc:MGI:2442413] | 0.65 | 6.10 | 0.039 |
| *Tia1* | cytotoxic granule-associated RNA binding protein 1 [Source:MGI Symbol;Acc:MGI:107914] | 0.59 | 6.08 | 0.035 |
| *Tial1* | Tia1 cytotoxic granule-associated RNA binding protein-like 1 [Source:MGI Symbol;Acc:MGI:107913] | 0.83 | 5.30 | 0.006 |
| *Tlr2* | toll-like receptor 2 [Source:MGI Symbol;Acc:MGI:1346060] | -0.70 | 3.65 | 0.043 |
| *Tnfaip3* | tumor necrosis factor alpha-induced protein 3 [Source:MGI Symbol;Acc:MGI:1196377] | -1.26 | 5.25 | 0.004 |
| *Tpr* | translocated promoter region nuclear basket protein [Source:MGI Symbol;Acc:MGI:1922066] | 0.60 | 7.81 | 0.046 |
| *Usp34* | ubiquitin specific peptidase 34 [Source:MGI Symbol;Acc:MGI:109473] | 0.67 | 7.76 | 0.021 |
| ***Vegfa*** | **vascular endothelial growth factor A [Source:MGI Symbol;Acc:MGI:103178]** | **-1.03** | **5.33** | **0.019** |
| *Xrn1* | 5'-3' exoribonuclease 1 [Source:MGI Symbol;Acc:MGI:891964] | 0.65 | 5.47 | 0.044 |
| *Ylpm1* | YLP motif containing 1 [Source:MGI Symbol;Acc:MGI:1926195] | 0.92 | 5.22 | 0.019 |
| *Zcchc11* | zinc finger CCHC domain containing 11 [Source:MGI Symbol;Acc:MGI:2445126] | 0.74 | 5.26 | 0.023 |
| *Zfp326* | zinc finger protein 326 [Source:MGI Symbol;Acc:MGI:1927246] | 0.63 | 4.15 | 0.039 |

**S1. Fig. S4. Original Cytoscape Network.** This is the original network It is supplied here to demonstrate that the Illustrator modification improve visualization, but did not alter the content of the network.

S1. Fig. S5. Cytoscape file. This file can be opened with Cytoscape, available from cytoscape.org
